# Mitochondrial targets for volatile anesthetics against cardiac ischemia-reperfusion injury

**DOI:** 10.3389/fphys.2014.00341

**Published:** 2014-09-16

**Authors:** Bhawana Agarwal, David F. Stowe, Ranjan K. Dash, Zeljko J. Bosnjak, Amadou K. S. Camara

**Affiliations:** ^1^Department of Anesthesiology, Medical College of WisconsinMilwaukee, WI, USA; ^2^Department of Physiology, Medical College of WisconsinMilwaukee, WI, USA; ^3^Cardiovascular Research Center, Medical College of WisconsinMilwaukee, WI, USA; ^4^Zablocki VA Medical CenterMilwaukee, WI, USA; ^5^Department of Biomedical Engineering, Marquette UniversityMilwaukee, WI, USA; ^6^Biotechnology and Bioengineering Center, Medical College of WisconsinMilwaukee, WI, USA

**Keywords:** volatile anesthetics, isoflurane, mitochondrial bioenergetics, electron transport chain, cardiac IR injury, cardioprotection

## Abstract

Mitochondria are critical modulators of cell function and are increasingly recognized as proximal sensors and effectors that ultimately determine the balance between cell survival and cell death. Volatile anesthetics (VA) are long known for their cardioprotective effects, as demonstrated by improved mitochondrial and cellular functions, and by reduced necrotic and apoptotic cell death during cardiac ischemia and reperfusion (IR) injury. The molecular mechanisms by which VA impart cardioprotection are still poorly understood. Because of the emerging role of mitochondria as therapeutic targets in diseases, including ischemic heart disease, it is important to know if VA-induced cytoprotective mechanisms are mediated at the mitochondrial level. In recent years, considerable evidence points to direct effects of VA on mitochondrial channel/transporter protein functions and electron transport chain (ETC) complexes as potential targets in mediating cardioprotection. This review furnishes an integrated overview of targets that VA impart on mitochondrial channels/transporters and ETC proteins that could provide a basis for cation regulation and homeostasis, mitochondrial bioenergetics, and reactive oxygen species (ROS) emission in redox signaling for cardiac cell protection during IR injury.

## Introduction

In recent years the mitochondrion has gained recognition as a key factor in the etiology of numerous diseases (Duchen, 2004), including cardiac ischemia and reperfusion (IR) injury (Ferrari, 1996; Murphy and Steenbergen, 2008b). Mitochondria act as critical triggers, mediators, and effectors in protective strategies directed against IR injury and other pathological situations (Camara et al., 2010, 2011). Cardioprotective strategies include a complex cascade of signaling events (Zaugg and Schaub, 2003) that not only involve the electron transport chain (ETC) but also key factors in the intrinsic anti-apoptotic signaling pathways that lead to cell protection. Consequently, mitochondria have emerged as regulators of the redox signaling, which is crucial in determining cell fate, i.e., life or death (Brookes et al., 2004).

**Figure 1:**
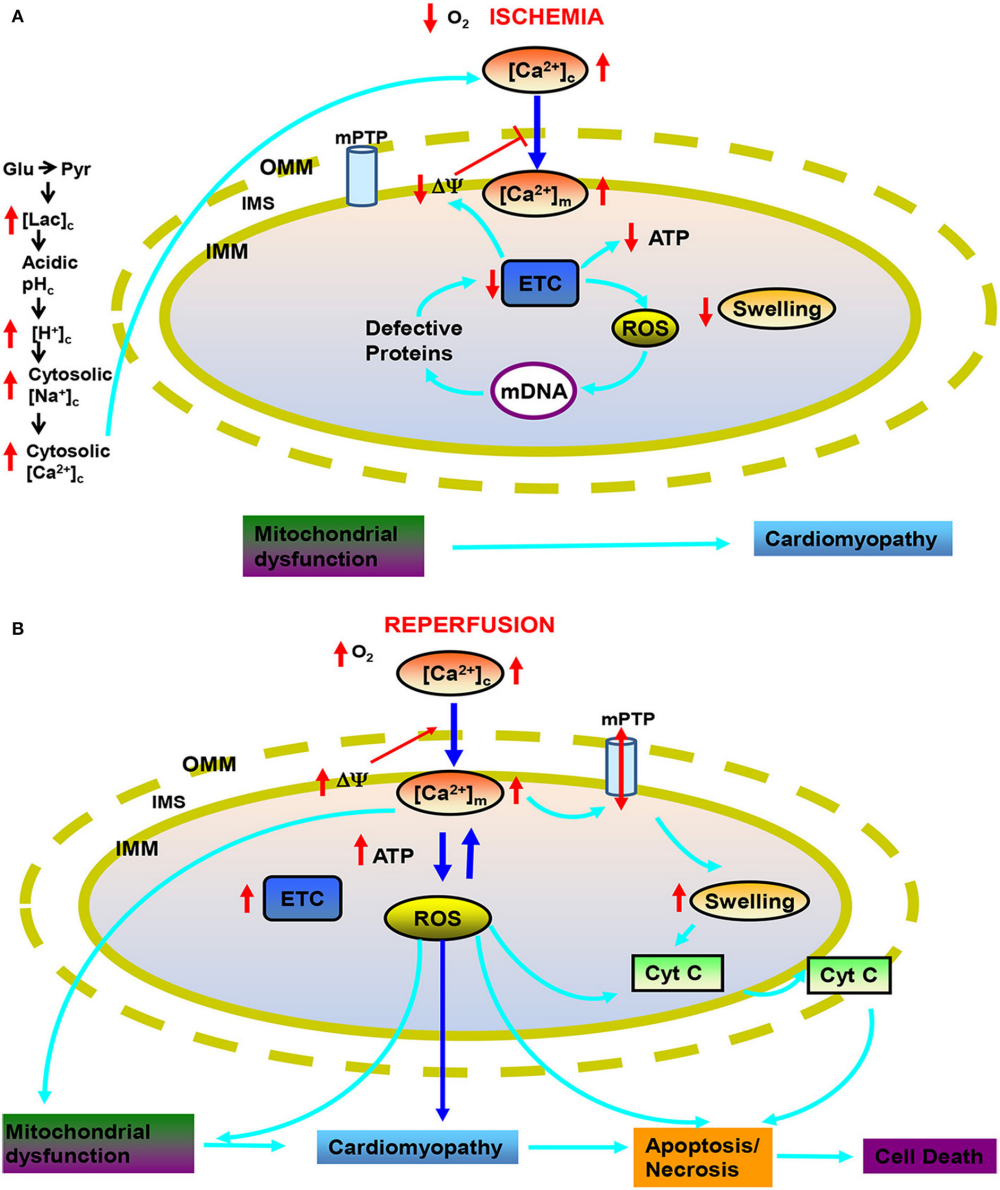
**Targets of mitochondria and sequence of changes in cytosolic and mitochondrial function during cardiac ischemia and reperfusion (IR) injury**. During ischemia **(A)** reduced O_2_ promotes anaerobic glycolysis that generates increased cytosolic lactate (lac_c_) leading to acidification. Increased H^+^ activates Na^+^-H^+^ exchanger (NHE) leading to increase cytosolic Na^+^ ([Na^+^]_c_), which activates Na^+^-Ca^2+^ exchanger (NCE), causing an increase in cytosolic Ca^2+^ ([Ca^2+^]_c_) which in turn increases mitochondrial matrix Ca^2+^ ([Ca^2+^]_m_). Impaired electron transport leads to increased generation of reactive oxygen species (ROS) beginning with superoxide (O^·−^_2_); impaired respiration and substrate utilization leads to uncoupling with lowered mitochondrial membrane potential (ΔΨ_m_) and decreased generation of mitochondrial ATP. During reperfusion **(B)**, the increase in deleterious ROS damages major macromolecules including tricarboxlic acid (TCA) enzymes, membrane transporters, electron transport chain (ETC) proteins and mitochondrial DNA (mtDNA). Also during reperfusion, ΔΨ_m_ is restored and [Ca^2+^]_m_ and ROS further increase to produce even greater mitochondria damage that induces mitochondrial permeability transition pore (mPTP) opening and release of cytochrome *c* (cyt C) that in turn triggers apoptosis. Other abbreviations: OMM, outer mitochondrial membrane; IMM, inter mitochondrial membrane; IMS, inter mitochondrial space.

Cardiac IR-induced mitochondrial dysfunction is accompanied by reduced membrane potential (ΔΨ_m_), decreased adenosine triphosphate (ATP) production, impaired Ca^2+^ homeostasis, increased “bad” reactive oxygen species (ROS) emission, matrix swelling and membrane permeability, and release of cytochrome *c* and other apoptotic factors leading to cell death (Steenbergen et al., 1990; Stowe and Camara, 2009) (Figure 1). Pre- and post-conditioning by volatile anesthetics (VA) have emerged as useful strategies to protect the myocardium against IR injury (Zaugg et al., 2003b; Pagel, 2008; Hu and Liu, 2009; Camara et al., 2010). Indeed, the guidelines of the American College of Cardiology and the American Heart Association recommend the maintenance of VA for non-cardiac surgery in patients with increased risk of myocardial ischemia (Fleisher et al., 2007). VA directly target many proteins to modulate their activities, which necessarily complicates analysis of their beneficial effects due to vague structural and dynamic consequences of VA interactions with their target proteins (Eckenhoff and Johansson, 1997). Also, despite advances noted in this review, the complete mitochondrial targets and mechanisms responsible for the protection afforded by VA remain unclear.

This review focuses primarily on the protein targets and functional effects of VA in mediating myocardial protection against IR injury. A special emphasis is given to the direct effects of VA on selected mitochondrial proteins and their implicated mitochondrial mechanisms for myocardial protection against IR injury. There are several cardioprotective strategies or treatments against IR injury directed to mitochondria (Krolikowski et al., 2005; Mewton et al., 2010; Chakrabarti et al., 2013; Jones et al., 2013). Indeed, the cardioprotective effects of VA, which likely include mitochondrial effects, have been tested clinically (Belhomme et al., 1999; Julier et al., 2003; Van der Linden et al., 2003; Zaugg et al., 2003a).

Bridging the gap between bench and bedside should be strengthened by unique therapeutic approaches against IR injury that are targeted to mitochondria. Indeed, because VA as a class are very lipophilic, unlike most other protective drugs, they readily penetrate mitochondria to target the more lipophilic protein sites embedded in the membrane structure. Thus by examining the role of lipophilic agents in mitochondrial-mediated cardioprotection, we may be able to define a new paradigm for mitochondrial protection that could lead to novel approaches to protect the heart in the clinical situation. We hope the information summarized here will provide helpful insights into the potential of synergistic effects of VA at multiple sites in mitochondria that underlie their cardioprotective effects.

## Molecular binding sites for VA

X-ray crystallography, molecular modeling, and structure–function studies indicate that anesthetics bind in hydrophobic cavities formed within proteins (Bertaccini et al., 2007). The lipophilic (or hydrophobic) nature of these binding sites underlies the Meyer–Overton correlation between anesthetic lipophilicity and potency (Hemmings, 2010). VA exhibit amphiphilicity (possessing both weak polar and nonpolar characteristics), which is required for effective interaction with these hydrophobic cavities, as indicated by a better Meyer–Overton correlation with more polar solvents (Hemmings, 2010). However, identification of anesthetic binding sites on any given target protein is quite difficult due to the low affinity interactions of VA and the paucity of atomic resolution structures for pharmacologically relevant target proteins like membrane bound proteins that are difficult to resolve structurally. In studies using albumin and luciferase, in which 3D atomic resolution structures are available, Bertaccini et al. (2007) found that VA bind in pockets with both nonpolar and polar non-covalent interactions. Binding involves weak hydrogen bond interactions with polar amino acid residues and water molecules, nonpolar van der Waals interactions, and a polarizing effect of the amphiphilic-binding cavity on the relatively hydrophobic anesthetic molecules (Hemmings, 2010).

Internal cavities underlie the conformational flexibility involved in ion channel gating and ligand-induced signal transduction of receptor proteins. Occupation of a critical volume within these cavities by VA provides a plausible mechanism for altering receptor and ion channel function by selective stabilization of a particular confirmation, e.g., an open or inactivated state of an ion channel. VA also acquire binding energy from the entropy generated by displacing bound water from these relatively promiscuous binding sites (Hemmings, 2010). Studies of glycine, GABA_A_, and NMDA receptors provide convincing evidence for the existence of anesthetic binding sites in critical neuronal signaling proteins by identifying the amino acid residues critical for anesthetic action (Wick et al., 1998; Koltchine et al., 1999; Jenkins et al., 2001; Hemmings, 2010). Although this review centers primarily on VA effects on cardiac mitochondrial protein activities that confer cardiac protection, it is expected that the molecular mechanism for VA action at mitochondrial sites are similar to those for other organelles.

## VA as pharmacological conditioning agents in IR injury

Clinically, myocardial ischemia (MI) is characterized by a reduced oxygen supply to demand ratio in the hearts of patients at high-risk of coronary artery disease (CAD) or who are undergoing high-risk cardiac surgery. Due to limited blood supply in the manifestation of MI, IR injury leads to a dysfunctional redox imbalance in mitochondria with a concomitant decrease in oxidative phosphorylation (OxPhos) and an overall switch to anaerobic metabolism. Experimental and clinical data have provided several types of mechanical and pharmacological conditioning strategies that lead to reduce IR-induced myocardial dysfunction and cell death as discussed next.

Murry et al. (1986) were the first to coin the concept of ischemic pre-conditioning (IPC), which involves adaptation of the myocardium to longer (damaging) IR stress when preceded by short episodes of repetitive ischemia and reperfusion. IPC evokes many downstream signaling factors (memory) to provide a lasting protection from subsequent lethal index ischemia. Many of the signaling kinases, including Akt/protein kinase B (PKB), protein kinase C-ε (PKC-ε), and extracellular regulated kinases (ERK1/2), translocate to mitochondria to contribute to the acute memory phase in cytoprotection against the impending index ischemia that can lead to heart damage (Zaugg and Schaub, 2003). Administration of a VA before myocardial IR as a protective strategy has been described in different animal and human models (Penta de Peppo et al., 1999; De Hert et al., 2002) as anesthetic-preconditioning (APC) (Tanaka et al., 2004a). APC invokes a memory phase by signaling kinases similar to IPC (Zaugg et al., 2003b). However, the detailed upstream mechanisms of mitochondrial-mediated protection by VA remain unclear.

Clinically, IPC can be mimicked pharmacologically by a variety of substances like aspirin, beta-blockers, alpha 2-adrenoceptor agonists, statins, opioids, and VA (isoflurane, halothane, desflurane, sevoflurane). Myocardial protection by the VA enflurane was first demonstrated by Freedman et al. (1985) in the isolated rat heart, global ischemia model. Later, Warltier et al. (1988) reported that halothane and isoflurane enhanced recovery of stunned myocardium in dogs during reperfusion. Novalija and Stowe (1998) reported that APC with sevoflurane mimicked IPC by improving vascular, mechanical, and metabolic function in isolated hearts through a sequence of molecular events that ultimately led to protection.

APC has two phases: an acute phase where the initial trigger phase of protection lasts for a few hours, and a delayed phase in which the protection is manifested days after washout of the VA. Although both acute and delayed APC are meditated through complex signal transduction cascades (Weber and Schlack, 2008), acute APC involves phosphorylation and translocation of preexisting proteins, while delayed APC involves *de novo* protein synthesis (Tonkovic-Capin et al., 2002; Tanaka et al., 2004b; Chiari et al., 2005b). APC shares major signaling events with IPC (Zaugg and Schaub, 2003; Zaugg et al., 2003b). That is, like IPC, APC enhances myocardial protection against infarction during early reperfusion by redox activation of protein kinases such as PI3K/Akt (as a part of the reperfusion injury salvage kinase (RISK) pathway), Pim-1 kinase [a member of the family of calcium/calmodulin-dependent protein kinases (CaMK II)], ERK1/2, and by glycogen synthase kinase (GSK-3β) dependent mechanism (Chiari et al., 2005a; Krolikowski et al., 2005, 2006; Weihrauch et al., 2005; Pagel et al., 2006; Stumpner et al., 2012b).

## The mitochondrion as a target for VA protection in IR injury

Mitochondria serve as the targets and end-effectors for a number of cellular metabolic processes including cell-signaling cascades, redox control, ion homeostasis, cell growth and cell death. In cardiomyocytes they are responsible for generating almost 95% of the cellular ATP; they are also responsible for the majority of the pathological ROS and reactive nitrogen species (RNS) produced during IR. VA likely interfere with mitochondrial function by directly or indirectly targeting many, but not all, mitochondrial proteins. Specifically, VA probably directly modulate the function of known targeted proteins that are believed to underlie the mechanism of IPC-induced protection against IR injury. Therefore, APC is of practical importance because administration of a VA could reduce mortality during ischemic heart surgery, and could also safely be given to patients who are at high risk, e.g., during heart transplant procedure (Ramakrishna et al., 2014). Although the mechanisms for this protection by APC are not well known, it is now evident that the mitochondrion is a key component in the beneficial effects of VA administration (Jovic et al., 2012; Mio et al., 2014).

**Figure 2:**
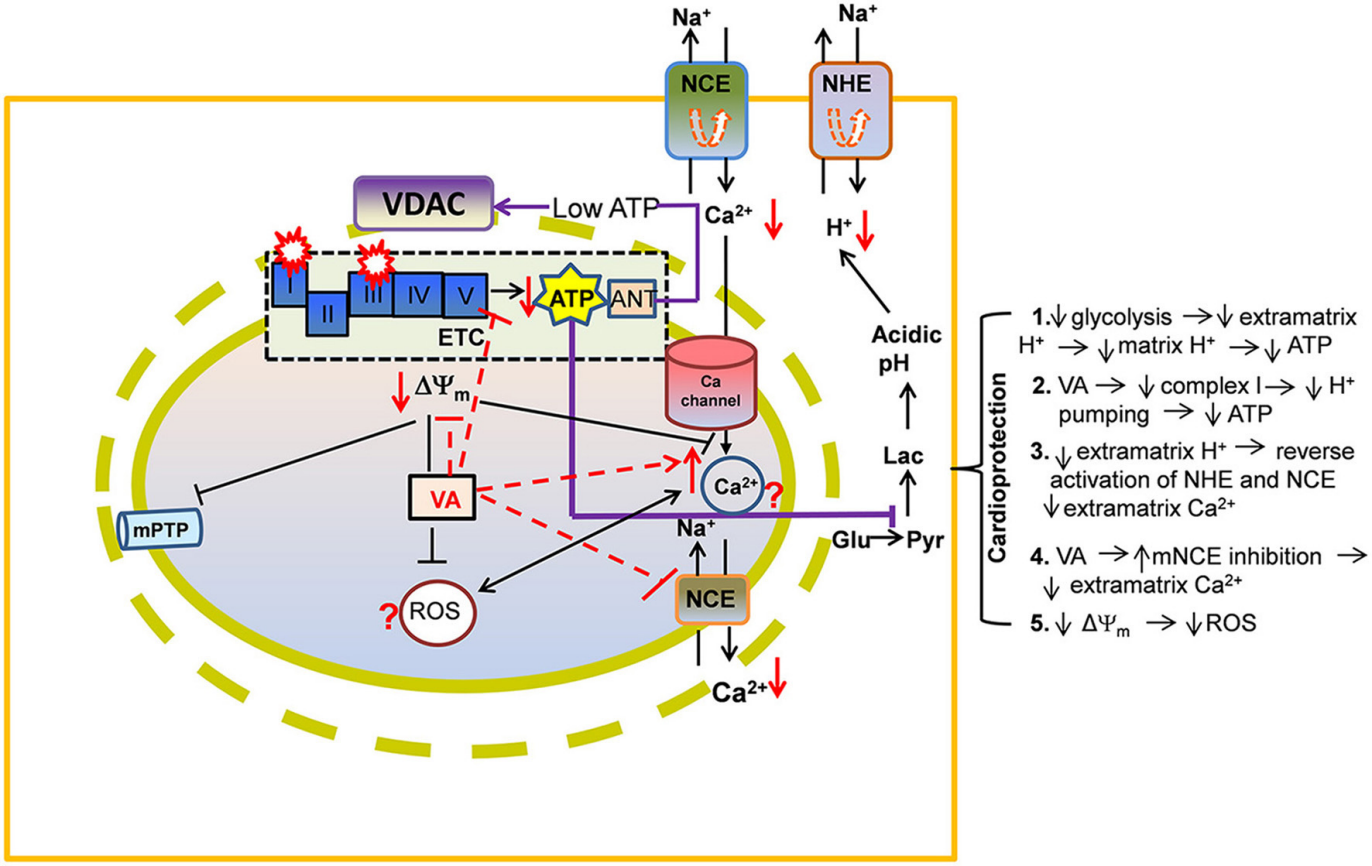
**A proposed view of cardioprotection by effects of volatile anesthetics (VA) on electron transport chain (ETC) proteins and on ADP/ATP transport via voltage-dependent anion channel (VDAC)**. By direct attenuation of NADH dehydrogenase (complex I) and cytochrome bc_1_ (complex III), VA promote a slightly more reduced redox state and a slowing of the rates of respiration and phosphorylation. Lowered ATP entry into the matrix through VDAC/ANT may contribute to reduced ATPase activity. These events may help to decrease ATP hydrolysis and so to better maintain cell ATP levels during reperfusion. Preserved ATP synthesis at complex V would diminish the need for glycolysis while decreasing lactic acidosis and cytosolic Ca^2+^ [Ca^2+^]_c_ (see details in Figure 1 legend). Other abbreviations: ROS, reactive oxygen species; mPTP, mitochondrial permeability transition pore; Symbol 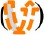 represents reverse functioning of NHE and NCE.

Based on its pharmacological effects, Kersten et al. (1996) reported that isoflurane-mediated protection against myocardial infarction in dogs involved the putative mitochondrial ATP sensitive K^+^-channel (mK_ATP_), discussed in detail later. VA appear to indirectly relax coronary arteries by altering intracellular Ca^2+^ regulation in the vascular smooth muscle cell by stimulating mK_ATP_ channel opening (Kersten et al., 1996) and/or opening of the mitochondrial big Ca^2+^ sensitive K^+^ channel (mBK_Ca_) (Redel et al., 2008). To date, potential VA-mediated cardioprotective mechanisms targeted to mitochondria involve inhibition of mitochondrial permeability transition pore (mPTP) opening (Pravdic et al., 2010; Sedlic et al., 2010b) (Figure 2) via activation of signaling kinases, like PKC (Novalija et al., 2003; Pravdic et al., 2009), mitochondrial uncoupling (Ljubkovic et al., 2007; Sedlic et al., 2009; Pravdic et al., 2012), “small” ROS emission (Tanaka et al., 2002; Novalija et al., 2003; Hirata et al., 2011), inhibition of mitochondrial Na^+^-Ca^2+^ exchange (mNCE) (Agarwal et al., 2012), modulation of mitochondrial bioenergetics (Sedlic et al., 2010a; Bienengraeber et al., 2013; Agarwal et al., 2014) (Figure 2), and opening of mK_ATP_ (Kersten et al., 1996, 1997; Pain et al., 2000; Stadnicka et al., 2006) and mBK_Ca_ channels (Ozcan et al., 2002; Stumpner et al., 2012a). These diffuse effects of VA on mitochondria may be attributed in part to the complex interactions of the mitochondrial proteins and their association with the membranes that separates the organelle from the cytoplasm and in part to the pleiotropic effects of VA on cell constituents. All of the activators may have a common final pathway, e.g., the triggering of a “small” amount of ROS to stimulate downstream protective pathways.

## Effects of VA on outer mitochondrial membrane proteins

The outer mitochondrial membrane (OMM) contains several enzymes including monomine oxidase and the integral transport proteins, porins, which makes the OMM permeable to small molecules less than 6 kilodaltons (kDa). The voltage-dependent anion channel (VDAC) constitutes the major porin of the OMM and it regulates the metabolic and energetic fluxes across the OMM by transporting metabolites and ions necessary for electron transfer, bioenergetics, and redox potentials for normal mitochondrial function. Mammalian mitochondria have three different VDAC isoforms: 1, 2, and 3 (Craigen and Graham, 2008) that perform different functions (Neumann et al., 2010). Recent reports also suggest a complex regulation of VDAC by mechanisms involving protein-protein interactions and post-translational modifications (PTMs) in normal and pathological conditions (Shimizu et al., 1999; Liu et al., 2009; Das et al., 2012; Porter et al., 2012; Yang et al., 2012). Effects of IR or other oxidative stresses can be exhibited by their ultimate actions on VDAC function. For example, De Stefani et al. (2012) postulated a mechanism by which VDAC permeability promotes apoptosis based on the close anatomic link between VDAC and the inositol triphosphate receptor (IP3) that transfers a large amount of Ca^2+^ from SR to mitochondria during cytosolic Ca^2+^ dysregulation.

The clinical relevance of VDAC in inducing apoptosis (Shoshan-Barmatz and Ben-Hail, 2012) indicates VDAC as a potential target for therapeutic drugs (Shimizu et al., 2001). The increased permeability of VDAC by VDAC oligomerization to create a large pore (Zalk et al., 2005) allows the release of apoptotic factors (e.g., cytochrome *c*), which activate proteolytic enzymes, e.g., the caspases. VDAC normally exists in the open configuration (Hodge and Colombini, 1997), whereas VDAC closure is associated with an increase in oxidative stress and increased Ca^2+^ dependent mPTP transition (Tikunov et al., 2010). The channel gating of VDAC from open to partial closure increased the Ca^2+^ permeability of VDAC (Rostovtseva et al., 2005) so Ca^2+^ imbalance may have a permissive role in mediating the mPTP transition. In contrast, drugs that prevent or impede VDAC closure could have potential therapeutic utility (Vander Heiden et al., 2000; Lemasters and Holmuhamedov, 2006). The modulation of VDAC permeability and release of cytochrome *c* is also regulated by other proteins, such as the Bcl-X protein family, the Bcl-2 homologous antigen/killer (Bak) and Bcl-2 associated X protein (Bax) (Shimizu et al., 1999); however, their definitive roles in pore size modulation is still debatable (Vander Heiden et al., 2000; Shimizu et al., 2001). The downregulation of Bcl-2 and upregulation of Bax protein in myocytes represents the molecular triggers and modulators of apoptotic cell death on reperfusion after ischemia (Zhao et al., 2000).

The anti-apoptotic protein Bcl-2 is also targeted to the mitochondrion and affects different mitochondrial metabolic functions (Imahashi et al., 2004). Isoflurane preconditioning was reported to block the myocardial IR induced decrease in the expression of anti-apoptotic Bcl-2 protein as well as the expression of the pro-apoptotic Bax protein; this led to an increase in the Bcl-2/Bax ratio, mediated through activation of PI3K/Akt signaling (Raphael et al., 2006). VA preconditioning was found to attenuate myocardial cell apoptosis in rabbits after regional IR via Akt signaling and modulation of Bcl-2 family proteins (Raphael et al., 2006). Overexpression of Bcl-2 reduced ischemic injury in hearts by affecting mitochondrial metabolic function as shown by a reduced rate of decline in ATP and enhanced acidification, consistent with Bcl-2 induced inhibition of consumption of glycolytically generated ATP (Imahashi et al., 2004). These effects could have been mediated by reduced entry of ATP into mitochondria via VDAC and/or adenosine nucleotide transport (ANT), or by direct inhibition of F_1_F_0_ ATPase. (Jamnicki-Abegg et al., 2005) (Figure 2) suggested that isoflurane reduces hypoxia-induced apoptosis through activation of Akt and by increased expression of anti-apoptotic Bcl-2 proteins. Thus, accumulating evidence points to a complex regulation of VDAC permeability/gating involving regulation by homo-oligomerization of VDAC, or by hetero-oligomerization with other mitochondrial proteins (e.g., ANT) and extra-mitochondrial proteins (Bak, Bax). Consequently, there are several potential targets for VA to exert their effects in the OMM that may reduce lethal permeabilization of VDAC and provide cardioprotection.

Evidence of VDAC regulation by GSK, the serine/threonine kinase family of proteins involved in glycogen metabolism, provides for an additional role of VDAC in cell injury during IR. Phosphorylation at Ser9 led to inhibition of GSK-3β during preconditioning and this was found to be cardioprotective against IR injury (Nishihara et al., 2006; Gross et al., 2008). The improvement in recovery of perfused rat hearts with a GSK-inhibitor was attributed to decreased ATP translocation through VDAC/ANT, or due to reduced ATP hydrolysis by F_1_F_0_-ATPase (Das et al., 2008); either pathway is consistent with decreased utilization of ATP as reported by Murry et al. (1986). The same mechanism of action was proposed to explain the noted improvement in post-ischemic recovery of mice hearts with overexpressed Bcl-2 (Chen et al., 2001). This was supported by another study that showed increased association of VDAC and Bcl-2 during ischemia (Imahashi et al., 2004). Further, the above mechanisms of GSK-dependent fall in ATP translocation into mitochondria was bolstered by a proteomic study that reported alterations in the expressions of ETC proteins during IPC using a GSK inhibitor (Wong et al., 2010) so it is interesting that isoflurane. Isoflurane-induced cardioprotection was also associated with increased levels of phosphorylation of GSK-3β (Zhu et al., 2010).

## Effects of VA on inner mitochondrial membrane proteins

**Figure 3:**
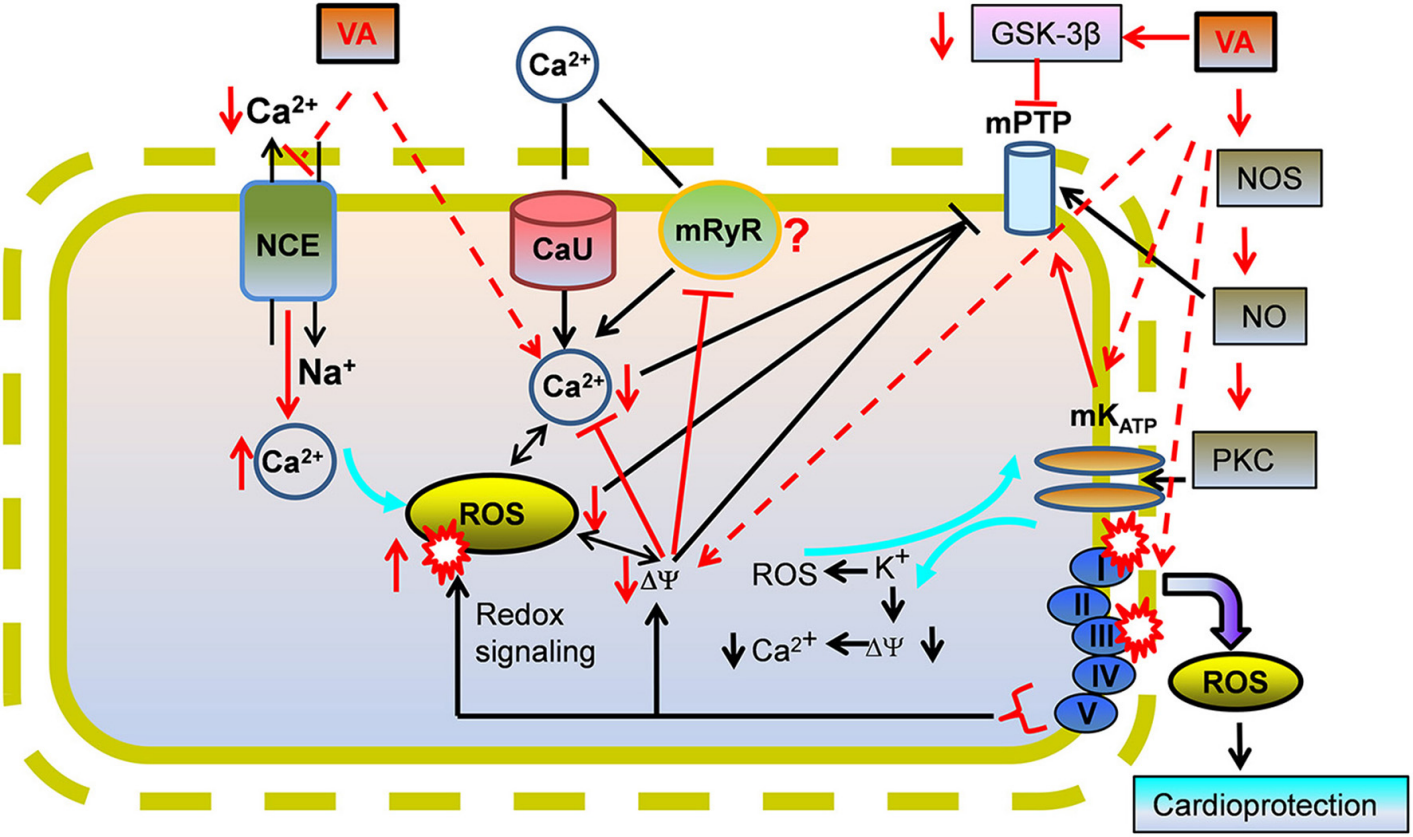
**A proposed view of cardioprotection by effects of volatile anesthetics (VA) on mitochondrial Ca^2+^ overload**. VA could mediate cardioprotection by mildly inhibiting mitochondrial NCE to increase [Ca^2+^]_m_ which triggers protective mechanisms before IR injury. Lowered ATP or higher Ca^2+^ -induced stimulation of mitochondrial K^+^ channels may lead to mild uncoupling by the K^+^-H^+^ exchanger (KHE) that may reduce ΔΨ_m_ and [Ca^2+^]_m_ during IR via the mitochondrial Ca^2+^ uniporter (CU) and/or the putative mitochondrial ryanodine receptor (mRyR). Lowered [Ca^2+^]_m_ would decrease “deleterious” ROS emission, impede mPTP opening, and reduce apoptotic/necrotic cell death on reperfusion. mPTP opening could also be prevented by a VA-mediated decrease in activation of glycogen synthase kinase (GSK-3β) via phosphorylation of GSK-3β. Effects of VA on channels/exchangers suggest potential implications for low Ca^2+^ and ROS in the triggering phase of VA cardioprotection.

The inner mitochondrial membrane (IMM) is impermeable to charged substances and so distinct channels, exchangers, and pumps are utilized to transport ions and metabolites to and from the matrix. The IMM also contains the ETC complexes that carry out OxPhos. This bioenergetic process is dependent on an intricate interplay among the supply of substrates, breakdown and elimination of metabolites, and ion fluxes across the IMM. For example, Ca^2+^ transport into and out of mitochondria is important for buffering excess cytosolic Ca^2+^ and for regulating mitochondrial respiration and ATP production to meet the cellular energetic demands, as in excitation-contraction coupling. Clearly, the mechanisms underlying myocardial contractile dysfunction during and after ischemic insults are due in part to impaired mitochondrial metabolism and ion homeostasis (Bosnjak and Kampine, 1986; Gerstenblith, 2004). A summary of IMM proteins that are affected by VA exposure and their implication in cardioprotection is given in Figure 3. VA-induced effects on respiratory complexes are discussed under “Mitochondrial bioenergetics as a target for VA.”

### VA and mitochondrial Ca^2+^ channels/transporters in IR injury

Myocardial IR leads to an increase in cytosolic [Ca^2+^], and consequently to mitochondrial Ca^2+^ loading (Steenbergen et al., 1987), which is a major contributor to mitochondria-mediated necrotic/apoptotic cell injury during IR. APC, like IPC, reduces cytoplasmic Ca^2+^ load and improves myocardial Ca^2+^ responsiveness so that reperfusion injury is attenuated (An et al., 2001). A detailed mechanistic understanding of this process remains to be explored. However, Riess et al. (2002b) reported that APC-mediated cardiac protection against Ca^2+^ overload on reperfusion was blocked by a putative mK_ATP_ inhibitor (5-hydroxydecanoic acid; 5-HD), suggesting that mK_ATP_ channel opening was associated with a decrease in matrix Ca^2+^ overload possibly via attenuated Ca^2+^ uptake.

The uptake of Ca^2+^ by the mitochondrial calcium uniporter (mCU) specifically depends on a large ΔΨ_m_ gradient (Saotome et al., 2005). Therefore, slight ΔΨ_m_ depolarization represents a strategy for cardioprotection. In a recent study we (Agarwal et al., 2012) found that direct exposure of mitochondria to isoflurane at a physiological Ca^2+^ concentration (~200 nM free) led to a Na^+^-dependent, but ΔΨ_m_-independent, increase in mitochondrial Ca^2+^ by attenuating NCE without affecting uptake via the mCU. Moreover, this was consistent with the lack of increase in matrix Ca^2+^ in the absence of buffer Na^+^ so that NCE could not be activated (Agarwal et al., 2012). These observations are compatible with a study by Sedlic et al. (2010b), in which a small increase in Ca^2+^ uptake was found in mitochondria isolated from isoflurane preconditioned rat hearts, even though mild loss of ΔΨ_m_ occurred. The decrease in ΔΨ_m_ also attenuated deleterious ROS production and attenuated mPTP opening. However, despite a fall in ΔΨ_m_ and a decrease in ROS emission, isolated mitochondria can still exhibit a small rise in matrix Ca^2+^, so there are exceptions to the idea that a rise in matrix Ca^2+^ is only a consequence of ROS-induced Ca^2+^ release phenomenon (Zima and Blatter, 2006). In our study (Agarwal et al., 2012), we speculated that the small increase in matrix Ca^2+^ induced by isoflurane could be part of the trigger mechanisms that include ROS in the signaling cascades that underlie VA cardioprotection. This notion remains to be verified experimentally. If proven, it could provide a novel insight into the triggering role of matrix Ca^2+^ in APC.

### VA and mitochondrial K_ATP_ (mK_ATP_) channels in IR injury

mK_ATP_ channels are thought to be located in the IMM, and like other mK^+^ channels with different ligands, are widely recognized as redox sensors of ischemia (e.g., low ATP, high Ca^2+^, low pH) that trigger effectors of several survival signaling pathways involved in pre- and post-conditioning (Gross and Fryer, 1999). In spite of numerous electrophysiological and pharmacological approaches to discern the molecular identity and composition of the mK_ATP_ channel, the true identity remains contentious. Using elaborate experimental approaches involving unbiased proteomic and pharmacological techniques, the KCNJ1 (ROMK) was identified in the IMM and demonstrated that ROMK channels can localize to mitochondria (Foster et al., 2012). The channel was shown to mediate ATP-sensitive K^+^ flux and to confer cytoprotection. However, as noted by Wojtovich et al. (2010) the assignment of ROMK as the mK_ATP_ conflicts with pharmacological data on the sensitivity of either channel to ATP and fluoxetine.

Nonetheless, mK_ATP_ channel opening, or any other mK^+^ channel, may be an important component of mitochondrial and cellular protection against cardiac IR injury. The cause-effect relationships of the components that lead to protection, however, are unclear. APC was reported to cause production of a small amount of ROS/RNS (Kevin et al., 2005), which could activate certain intracellular signals, like NO^·^, that led to activation of the mK_ATP_ channel (Novalija et al., 1999). Putative mK_ATP_ channel openers led to mild swelling and uncoupling of mitochondria (mild loss of ΔΨ_m_), and a “small” transient rise in ROS emission (signaling ROS) associated with a decrease in mitochondrial Ca^2+^ load (Wang et al., 2001; Facundo et al., 2006a,b). VA exert cardioprotective effects that most certainly involve mitochondrial bioenergetics (discussed later), and also mK_ATP_ channel (or other K^+^ channels) opening, as reviewed by our group previously (Riess et al., 2004b; Stowe and Kevin, 2004; De Hert et al., 2005; Kevin et al., 2005).

Several other reports support the association of VA and K_ATP_ channels on mitochondrial function. Jiang et al. (2007) reconstituted fragments of the IMM from human left ventricle, and based on use of the putative mK_ATP_ channel antagonist, 5-HD, they reported that isoflurane increased the open probability of the putative mK_ATP_ channel. Similarly, H_2_O_2_ was able to activate the putative mK_ATP_ channel; this finding was supported by a similar study (Queliconi et al., 2011). These data confirm that isoflurane, as well as ROS, directly modulate reconstituted cardiac mK_ATP_ channels without apparent involvement of cytosolic protein kinases, as commonly proposed. Sevoflurane preconditioning protected the myocardium against IR injury by reducing mitochondrial Ca^2+^ loading, again presumably via mK_ATP_ channel opening (Wang et al., 2001; Chen et al., 2002; Liu et al., 2005). Sevoflurane induced cardioprotection was also proposed to increase mitochondrial volume via mK_ATP_ channel opening on the basis of effects of putative agonists and antagonists on K_ATP_ channels (Riess et al., 2008b). Desflurane prevented mPTP opening and this effect was abrogated by pretreatment with a mK_ATP_ channel antagonist, which suggested a link between mPTP opening and mK_ATP_ channel activation during cardioprotection (Riess et al., 2002b, 2003, 2008a; Piriou et al., 2004). However, the mechanism regulating mK_ATP_ dependent mPTP transitions still remains to be verified. Moreover, the sensitivity and selectivity of 5-HD and diazoxide as modulators of mK_ATP_ channels have been questioned (Hanley et al., 2002; Lim et al., 2002; O'Rourke, 2004). Thus, although these studies suggest overall that VA act on mK_ATP_ channels as a mechanism to contribute to cell protection, the effects could have been on other mK^+^ channels or due to other upstream mechanisms.

### VA and mitochondrial permeability transition pore (mPTP) in IR injury

The mPTP is a non-specific channel that allows water, ions, and solutes with low molecular weights (≤1.5 kDa) to traverse mitochondrial membranes and enhance ROS emission, mitochondrial swelling and cell death. The molecular identity of the mPTP remains unclear. The mitochondrial matrix protein, cyclophilin D (CyP-D), a member of a family of highly homologous peptidylprolyl cis-trans isomerase (PPIase), is believed to constitute an integral component of the mPTP, and thus to play an important role in regulating the pore (Nicolli et al., 1996). Previously it was suggested that the VDAC-ANT-CyP-D complex constituted the structural and functional component of the mPTP by its sensitivity to cyclosporin A (CsA), an inhibitor of the pore (Crompton et al., 1998). However, subsequent genetic loss- and gain-of-functional studies have questioned the relevance of VDAC in the formation of mPTP (Javadov et al., 2009; Bernardi, 2013). Interestingly, in a recent study, the F_1_F_0_-ATP synthase was proposed to be the mPTP, or at least a component of the mPTP complex (Giorgio et al., 2013). Increased mitochondrial ROS, in addition to Ca^2+^ overload, alkalosis, and ATP depletion (Halestrap, 2010), are major hallmarks of IR injury and are some of the primary factors that lead to mPTP opening.

IR-activated pathways of cell death are likely mediated by mPTP because CsA and sanglifehrin A, inhibitors of the pore, were found to reduce infarct size (Clarke et al., 2002). Thus, preventing mPTP opening serves as a clinically relevant therapeutic target for treating IR injury. VA-induced cardioprotection is associated with reduced mPTP opening (Krolikowski et al., 2005; Pravdic et al., 2009; Sedlic et al., 2010b). NO^·^ produced by endothelial NO^·^ synthase (eNOS) during cardioprotection by anesthetic post-conditioning (APoC) was suggested to prevent mPTP opening (Ge et al., 2010). Sevoflurane, like CsA, increased the threshold of Ca^2+^-induced mPTP opening when mediated via GSK-3β inactivation (Onishi et al., 2012). Moreover, the interaction of PKC-ε and the putative constituents of the pore (VDAC, ANT) suggested that a signaling mechanism could modulate mPTP function (Baines et al., 2003). Isoflurane preconditioning reduced cytochrome *c* release (Qian et al., 2005), possibly by activating a PKC-3-dependent mechanism linked to retarded mPTP opening. Isoflurane was found to induce phosphorylation of GSK-3β, which was associated with mitochondrial protection and reduced IR injury due to attenuated mPTP opening (Juhaszova et al., 2004). Moreover, phosphorylation of GSK-3β was reported to increase binding of ANT with phosphorylated GSK-3β (Nishihara et al., 2007), which decreased binding of ANT with CyP-D and suppressed mPTP formation to ultimately confer cardioprotection (Hausenloy et al., 2002; Javadov et al., 2003). Future confirmation of the molecular identity of mPTP is indispensable to understanding VA-mediated mechanisms that would potentially retard mPTP opening and confer cardioprotection. An isoflurane-mediated decrease in ROS production inhibited earlier opening of the mPTP and reduced apoptosis (Wu et al., 2014) during hypoxia/reoxgenation in isolated cardiomyocytes.

## Mitochondrial bioenergetics as a target for VA

### Function of mitochondrial electron transport chain (ETC) complexes

**Figure 4:**
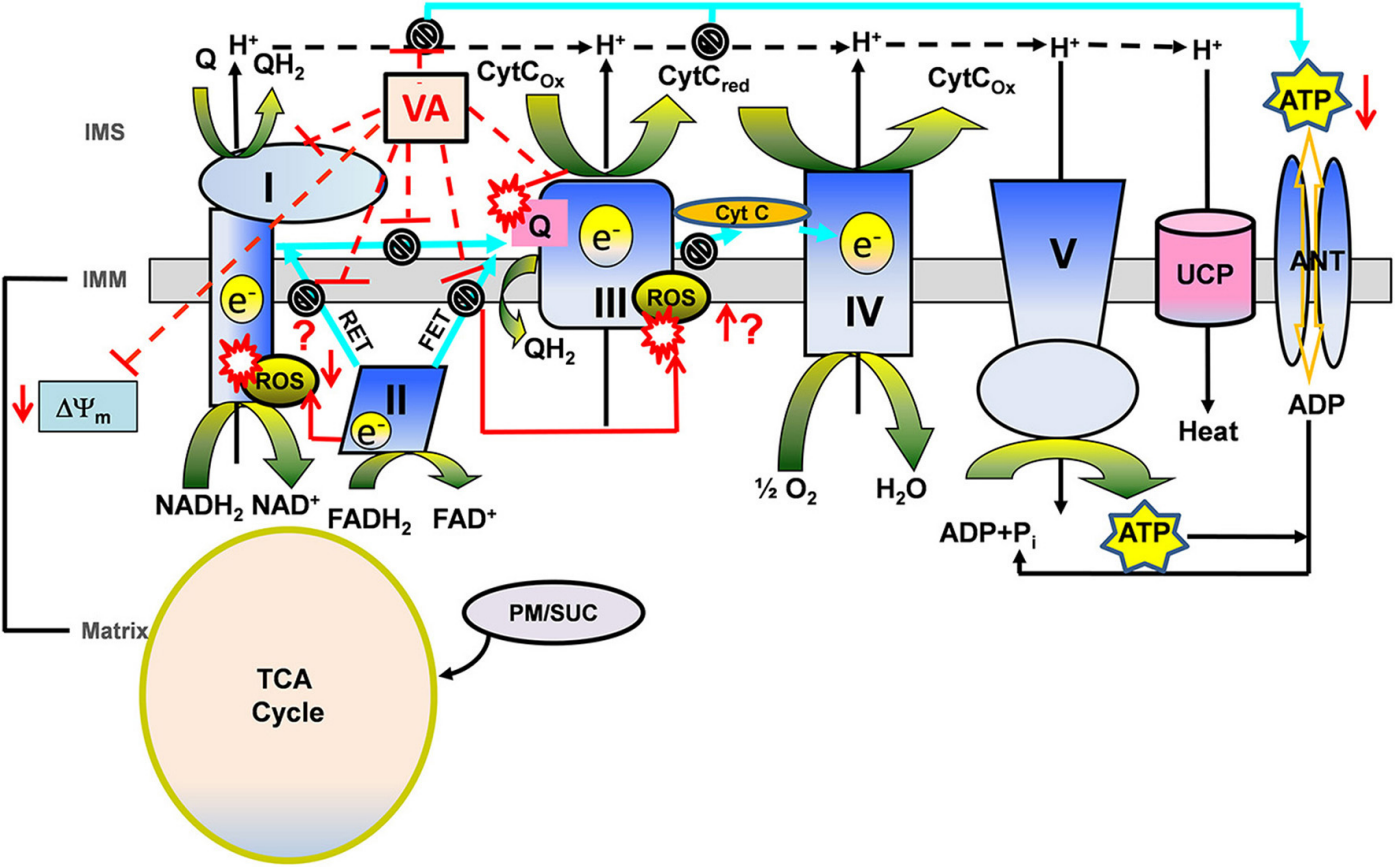
**A proposed view of cardioprotection based on the modulating effects of volatile anesthetics (VA) on electron transport chain (ETC) function in the presence of different substrates**. Mitochondria generate reducing equivalents, NADH_2_ and FADH_2_, via the tricarboxylic acid (TCA) cycle in the matrix during electron transfer through the ETC complexes of the inner mitochondrial membrane (IMM) to generate the proton gradient (H^+^) and mitochondrial membrane potential (ΔΨ_m_). The transfer of electrons through the ETC complexes to the final electron acceptor, O_2_, is coupled with the H^+^ gradient to phosphorylate ADP to ATP by ATP synthase (complex V). During electron transfer, attenuation of complex I and complex III generates O_2_ free radical anions (O^·−^_2_) leading to other ROS. Complex II mediates forward and reverse electron transfer (FET and RET, respectively), which generates ROS at complex I and complex III. VA modulate complex I and III, therefore affecting bioenergetics (ΔΨ_m_, redox state, respiration, phosphorylation). During oxidation of complex II substrate succinate (Suc), ROS generated via RET may be considered “deleterious,” while ROS generated via FET by complex III inhibition may represent the “signaling” ROS. VA-induced complex I inhibition may decrease the generation of RET mediated “deleterious” ROS and could mediate the generation of “signaling” ROS at complex III. Whereas, during oxidation of complex I substrate pyruvate/malate (PM), VA-induced complex I inhibition could mediate generation of “signaling” ROS at complex I. VA-induced complex I and complex III inhibition may mediate slower rates of proton and electron transfer to reduce ATP synthesis during ischemia to preserve it during reperfusion. Possible modulating effects of VA on uncoupling protein (UCP) in promoting proton leak and uncoupling in cardioprotection are also noted. Other abbreviations: ANT, adenine nucleotide translocase; TCA cycle, tricarboxylic acid cycle; IMS, inter mitochondrial space.

Mitochondria regulate metabolism in addition to synthesizing ATP. Mitochondrial dysfunction underlies various pathological processes, including IR injury, as emphasized in this review. Consequently, preservation of mitochondrial function is necessary to abrogate mitochondrial energy imbalances and apoptotic signaling pathways that occur in IR injury (Chen et al., 2007). Delineating the underlying molecular mechanisms that act either as triggers, activators, or end-effectors is crucial for understanding the complex cardiac protective vs. detrimental mechanisms mediated by mitochondria. An understanding of how VA alter mitochondrial bioenergetics is highly significant because mitochondrial respiratory dysfunction is reportedly a trigger of IR injury and VA are cardioprotective. The scheme representing known VA targets of ETC proteins and their modulating effects on ETC functions are summarized in Figure 4.

VA are well known to mediate myocardial protection in part by attenuating the activity of ETC proteins, the first of which is complex I (Riess et al., 2002a, 2005). Attenuating activity of complex I appears to produce a small transient increase in ROS, which could then serve as a trigger for cellular protection (Kevin et al., 2003; Riess et al., 2004a). During oxidation of complex I substrate, complex III is considered the principal source for ROS generation in isolated mitochondria; but this can be inhibited by limiting electron flow from complex I to complex III (Chen et al., 2003; Aldakkak et al., 2008). A sevoflurane-mediated decrease in complex I activity was reversed with ROS scavengers; this suggested that the trigger in protection involves modulation of ETC complexes and generation of ROS (Riess et al., 2004a).

In our most recent study (Agarwal et al., 2014) we explored the potential ETC protein targets of isoflurane by comparing its effects with known ETC inhibitors. We found a differential modulation of NADH, ΔΨ_m_, and respiration by isoflurane under different substrates conditions. We furnished inferential evidence that isoflurane directly attenuates forward and reverse electron flow, in a substrate dependent manner, by selectively inhibiting ETC complexes I and III. Complexes II, IV, and V, as well as ANT activities were unaffected by isoflurane. These results supported some selectivity of isoflurane in its interaction with different mitochondrial proteins. With the complex I substrate, pyruvate/malate, isoflurane decreased the magnitude of state 3 NADH oxidation, increased transient state 3 depolarization, and depressed state 3 respiration by attenuating complex I in a manner similar to low concentrations (nM) of a complex I inhibitor (rotenone).

Limiting complex I activity during ischemia has the potential to minimize ROS accumulation on reperfusion and so to protect mitochondria and cells from oxidative damage (Aldakkak et al., 2008). With the complex II substrate succinate, isoflurane only slightly reduced NADH oxidation, ΔΨ_m_ depolarization and state 3 respiration (Agarwal et al., 2014). In the presence of succinate and inhibition of complex I with rotenone, isoflurane increased the rates of state 3 and 4 respiration by attenuating complex III activity. Attenuated electron transfer at complex III leads to electron leak and ROS generation. Thus, the cardioprotective effect of VA against IR injury could be triggered by a small rise in ROS, which can occur with modulation of the activity of ETC complexes. This is supported by a study (Ludwig et al., 2004a), in which an isoflurane-induced small increase in ROS and reduction in myocardial infarct size *in vivo* were attenuated by a complex III inhibitor, but not by a complex I inhibitor. This study suggested that ROS generation from complex III at some point during IR injury is a crucial intracellular redox mediator of isoflurane-induced preconditioning.

The generation of a “small” transient signaling ROS from the ETC most likely originates from the ubisemiquinone (Q^·^) radical intermediate via electron transport in complex III (Chen et al., 2003) and so this may be a crucial mediator of VA-induced conditioning (Kevin et al., 2003; Ludwig et al., 2004a). Hirata et al. (2011) reported that isoflurane increased the generation of signaling ROS at complexes I and III, and decreased the reverse electron flow -mediated detrimental ROS generation, by attenuating complex I activity during reperfusion. Thus, attenuation of mitochondrial complexes I and III by VA may trigger the signaling ROS that, via downstream pathways, decrease production of deleterious ROS from these complexes and confer cardioprotection. In the isolated heart model, APC also may be triggered by the formation of a small transient amount of RNS, because decreased cardioprotection was found during VA exposure with application of either a ROS scavenger or a NO^·^ inhibitor (Novalija et al., 2002). VA also enhanced myocardial recovery during reperfusion by opposing the adverse effects of deleterious ROS on cardiac function (Tanguay et al., 1991). It is also possible that aside from the direct modulatory effects of VA on ETC complexes that leads to slight increase in the “triggering” ROS production, VA could mediate their effects on ROS generation through modulation of the ROS balance, i.e., generation vs. scavenging, by affecting the mitochondrial antioxidant defense mechanism (Nickel et al., 2014).

### VA and mitochondrial membrane potential

The active pumping of protons (H^+^) from the matrix to the inter-membrane space generates the ΔΨ_m_ necessary for OxPhos. A H^+^ leak (uncoupling) in the IMM has potential implications in both IR injury and in preconditioning (Nadtochiy et al., 2006). Murphy et al. (2003) suggested that exposure to ROS mediates activation of uncoupling proteins (UCPs), which tend to reduce ΔΨ_m_ and hasten respiration. A lowered mitochondrial pH and ΔΨ_m_ may be markers of VA-induced cardioprotection (Pravdic et al., 2010). The direct effect of isoflurane to decrease ΔΨ_m_ due to reduced complex I activity and increased mitochondrial acidification via an ATP synthase-mediated increase in proton influx (Pravdic et al., 2012) is another alternative mechanism for VA-induced cardioprotection. Preconditioning may increase ROS production due to flavoprotein oxidation and mitochondrial uncoupling while the decrease in ΔΨ_m_ may be coupled to mK_ATP_ channel opening (Ljubkovic et al., 2007; Sedlic et al., 2009).

Sedlic et al. (2010a) examined isoflurane's site of action in the ETC using an isolated rat cardiomyocyte model and reported an uncoupling-induced depression of ΔΨ_m_ and complex I inhibition by isoflurane as two potential mechanisms contributing to protection against IR injury. It was reported that UCP-3 protected the heart against IR injury and that UPC-3 knockout mice lost the cardioprotection conferred by IPC (Ozcan et al., 2013). However, Pravdic et al. (2012) reported that isoflurane, like UCPs, caused a mild depolarization and matrix acidification possibly by reducing complex I function and increasing H^+^ flux through ATP synthase; they also reported that UCPs appeared not to be involved in APC. A decrease in ΔΨ_m_ and reduced mitochondrial Ca^2+^ uptake, with concomitant tolerance to hypoxia-reoxygenation, was reported to occur in isolated cardiomyocytes and mitochondria examined after cardiac preconditioning in rats (Ljubkovic et al., 2007). In another study, an APC-induced decrease in ΔΨ_m_ was reported to be beneficial in decreasing excess ROS emission and mitochondrial Ca^2+^ accumulation in oxidatively stressed cardiomyocytes; this effect was suggested to be due to a mild uncoupling effect (Sedlic et al., 2010b). However, in many of these studies the physiological relevance of mild uncoupling by VA, if it occurred, is questionable (Shabalina and Nedergaard, 2011) because the depression of ΔΨ_m_ was observed only in isolated mitochondria metabolizing a high concentration of succinate (Shabalina and Nedergaard, 2011) and exhibiting a large increase in respiration (Agarwal et al., 2014). Moreover, it is possible that the mild uncoupling was mediated by an effect of VA to activate other mK^+^ channels. In any case, the molecular basis of VA interactions with the mitochondrial ETC proteins that leads to decreased or unchanged ΔΨ_m_ is remains unclear.

Oxidative phosphorylation is central to substrate metabolism and energy production. Wide variations in OxPhos rates occur to match workload demand to ATP supply. This rate is regulated in part by Ca^2+^-induced activation of several TCA enzymes; but other factors are also involved (Vinnakota et al., 2011; Boelens et al., 2013). On reperfusion, APoC was shown to depress mitochondrial respiration, to partially depolarize mitochondria, and to decrease mitochondrial pH (Pravdic et al., 2010). These events led to retarded mPTP opening and, consequently to better preserved ΔΨ_m_ and ATP synthesis, and reduced intracellular and mitochondrial Ca^2+^ overload and cell death (Pravdic et al., 2010). Isoflurane pre- and post-conditioning was reported to induce phosphorylation of mitochondrial proteins, with ANT phosphorylation as a novel mitochondrial therapeutic strategy for IR injury that could confer protection by preventing the ischemic-induced dephosphorylation of ANT (Feng et al., 2008). The coordinated expression of two genomes, nuclear and mitochondrial, regulates the biogenesis of OxPhos (Garesse and Vallejo, 2001). One report suggested that sevoflurane induced delayed conditioning by activating nuclear factor-κB (NF-κB) (Qiao et al., 2013), an inducible transcription factor produced in response to ROS and RNS, and that this modulation could help control the transcription of DNA and cellular responses to stress stimuli against myocardial injury by limiting apoptosis.

## Metabolic role of VA in cardioprotection

Mitochondria normally generate ATP by electron transport, H^+^ pumping and OxPhos. But during ischemia the shortage in substrates and O_2_ decreases OxPhos promoting the working of F_1_F_0_ ATPase in reverse. Reduced cellular ATP levels stimulate glycolysis causing lactic acidosis and a rise in intracellular Na^+^ by activating the Na^+^-H^+^ exchanger (NHE). In turn this leads to an increase in intracellular Ca^2+^ by activating the sarcolemmal Na^+^-Ca^2+^ exchanger (NCE) (Murphy and Steenbergen, 2008a), as shown in Figure 2. An alteration in mitochondrial membrane transport protein function, e.g., VDAC, can contribute to IR injury by impeding delivery of substrates required to carry out OxPhos. In an overview of VDAC functional regulation in cell death during cardiac IR, Das et al. (2012) suggested that less entry of nucleotides via VDAC during IR injury might protect cells by reducing the rate of ATP utilization. A decline in cell ATP utilization during ischemia is considered fundamental for cardioprotection in an IPC setting by improving ATP availability during reperfusion (Murry et al., 1986). Thus, a decrease in ATP production and reduced ATP entry into mitochondria through VDAC could lead to reduced ATP consumption by F_1_F_0_-ATPase, and to reduced glycolysis and lactic acidosis, which ultimately could lead to decreased cytosolic Ca^2+^ loading via cation exchangers (Murphy and Steenbergen, 2008a). A reduction in glycolysis also leads to decreased cytosolic H^+^ and less H^+^ entry into mitochondria.

The studies above indicate that alterations in mitochondrial membrane proteins play crucial roles in indirectly modulating mitochondrial Ca^2+^ overload and excess ROS emission. Indeed, modulation of VDAC function by VA, directly or indirectly, as reported by Jamnicki-Abegg et al. (2005), Raphael et al. (2006), and Zhu et al. (2010), may reduce the vicious feed-forward cycle of Ca^2+^ overload and ROS emission that culminates in cell demise. Given the numerous roles of VDAC in transfer of anion/cation and other metabolites, there could be additional mechanisms involving mitochondrial membrane proteins-induced cardioprotection and any role of VA in these complex mechanisms have yet to be explored.

## Effects of VA on signal transduction pathways during IR injury

Exposure to VA preceding IR leads to activation of several signaling cascades that involve protein kinases and “small” transient ROS/RNS, including NO^·^. These signaling molecules eventually converge on mitochondria to provide protection (Zaugg et al., 2003b; Walters et al., 2012). Marinovic et al. (2006) provided evidence in support of a dual role mK_ATP_ channels in VA protection, i.e., as a trigger to initiate the signaling cascade and as an effector responsible for the cardioprotective memory. APC with sevoflurane was reported to improve vascular and mechanical function by increasing NO^·^ release that was blocked by an mK_ATP_ channel inhibitor (Novalija et al., 1999). In an *in vivo* rat model, Ludwig et al. (2004b) suggested that APC is mediated by opening of mK_ATP_ channels and the subsequent generation of transient ROS, which activates protein kinases. In another study, Pravdic et al. (2009) inferred that APC is mediated by a PKC-δ-induced delay of mPTP opening. Lastly, another study showed that APC protected the mouse heart against reperfusion injury by preventing mPTP opening in an eNOS dependent manner, with NO^•^ acting as both the trigger and the mediator of cardioprotection (Ge et al., 2010).

The G protein coupled receptor 30 (GPR 30) agonist G1 improved cardiac function, reduced infarct size, and inhibited mPTP opening by activating ERK signaling in the isolated mouse hearts after IR (Bopassa et al., 2010). The pro-survival kinases ERK1/2 and PI3K/Akt also appear to contribute to VA mediated cardioprotection (Raphael et al., 2005; Wang et al., 2006b). Heat shock protein 90 (HSP 90), a cytoprotective protein, facilitated the translocation of PKC-ε after IR, and increased phosphorylation of mitochondrial aldehyde dehydrogenase 2 (ALDH2) (Budas et al., 2010). ALDH2 is known to detoxify 4-hydroxynonenal (HNE), a cytotoxic end product of lipid peroxides following oxidative stress, by oxidizing the aldehyde group (Camara et al., 2010). In this case, increased ALDH2 activity resulted in reduced cardiac injury in an animal model of myocardial infarction (Budas et al., 2010). A recent study showed that isoflurane-induced APC alleviated hypoxia-reoxygenation injury in conjunction with PKC-δ mediated activation of mitochondrial ALDH2 (Lang et al., 2013). Bouwman et al. (2006) reported that activation of PKC-δ by sevoflurane increased sarcolemmal NCE mediated myocardial Ca^2+^ influx, which may be a trigger of cardioprotective signaling events during APC. Desflurane preconditioning was reported to activate BK_Ca_ channels through protein kinase A (PKA) (Redel et al., 2008). Exposure to isoflurane during early reperfusion induced cardioprotection associated with increased expression of the anti-apoptotic Bcl-2, a modulator of mPTP (Wang et al., 2006a). Isoflurane also protected hearts from IR injury, possibly by preventing excess ROS generation and mPTP opening that in turn inhibited the activation of caspase-3 (Wu et al., 2014). Although the signaling pathways are very complex and incompletely resolved, it is likely that VA modulate other known and unknown mitochondrial channel/transporters involved in IR injury; but this remains to be tested.

## Other potential mitochondrial targets of VA during IR injury

### Post-translational modifications

Beneficial post-translational modifications (PTMs) of mitochondrial proteins have been proposed to modulate cardioprotection (Foster et al., 2009; Pagliaro et al., 2011; Porter et al., 2012). A modification of mitochondrial protein by O-glycosylation with O-linked-β-N-acetyl glucosamine (O-GlcNAc) was suggested to occur with IPC as assessed by improved cardiac myocyte survival due to attenuated ΔΨ_m_ (Jones et al., 2008). In a recent study (Champattanachai et al., 2008) it was reported that the protection by increased O-GlcNAc during injury of neonatal rat ventricular myocytes was mediated by enhanced mitochondrial Bcl-2 translocation. *In vivo* and *ex vivo* studies with isoflurane preconditioning in mice demonstrated increased O-glycosylation of cardiac mitochondrial VDAC associated with resistance to IR stress (Hirose et al., 2011).

VA-mediated PTM (mostly phosphorylation) of mitochondrial proteins involved in bioenergetics and electron transport complexes are implicated in the role of PTMs in regulating mitochondrial function that confers cardioprotection (Arrell et al., 2006; Kalenka et al., 2006; Wong et al., 2010). However, additional studies are needed to validate the functional effects of these changes during the various conditioning periods against IR injury. Signaling RNS can also induce beneficial, reversible PTMs. Specifically, S-nitrosylation of some mitochondrial proteins may lead to cardioprotection during IPC and IPoC (Tullio et al., 2013). As noted before, complex I dysfunction resulting from oxidative damage is an important factor in the pathogenesis of IR injury (Murray et al., 2003). Therefore, another possible mechanism of cardioprotection is modulation of complex I protein by NO^·^-induced S-nitrosation leading to beneficial modulation of bioenergetics and redox signaling (Burwell et al., 2006). Complex IV is another target of NO^·^ where it competes with O_2_ at its binding site (Brookes et al., 2001); Similarly, VA were also reported to modulate complex IV activity and to alter its function (Casanovas et al., 1983; Szabo and Zoratti, 1993); however, whether this was through NO^·^ was not evident. As noted earlier, in our recent study (Agarwal et al., 2014), we did not observe an effect of isoflurane on complex IV function. This discrepancy in VA modulation of mitochondrial function as a cardioprotective strategy further supports the complexity of VA interaction with mitochondrial proteins.

Recently, changes in the mitochondrial proteome during APC were assessed by a proteomic mass spectral approach (Bienengraeber et al., 2013). An ^18^O-labeling method was applied to relatively compare cardiac mitochondrial samples from control and isoflurane exposed rats before and after IR. It was found that the activities of ATP synthase, a complex I subunit, citrate synthase, and isocitrate dehydrogenase were increased after APC compared to IR only based on phosphorylation of the proteins (Bienengraeber et al., 2013). Since, those modulated proteins directly belong to the OxPhos system, these observations further confirm the role of VA in altering mitochondrial bioenergetics/metabolism.

### MicroRNAs

miRNAs are endogenous, small non-coding, single stranded RNAs (ssRNAs, ~22 nucleotides) that are involved in transcriptional and post-transcriptional regulation of gene expression (Chen and Rajewsky, 2007). Several recent reports suggest miRNAs are novel therapeutic biomarkers for IR injury (Cheng et al., 2010a), but their potential application in myocardial protection against IR is not known. The up- or down-regulation of miRNAs have been reported to occur during IR; in particular, protective effects of miRNAs with their target genes were identified that reduced cardiac cell apoptosis during pre- and post- conditioning against cardiac IR injury (Dong et al., 2009; Cheng et al., 2010b; He et al., 2011). One study reported that upregulation of miRNAs was involved in delayed preconditioning, in which miRNAs appeared to upregulate proteins (eNOS, HSP70) involved in delayed preconditioning after IPC (Yin et al., 2009). The role of miRNAs in APC or as a direct target of VA has not been reported. However, according to a recent preliminary report by Olson et al. (2013), *in vitro* application of isoflurane caused upregulation of miR-21 and conferred cardioprotection, while knockdown of miR-21 attenuated cardioprotection. Moreover, in that study attenuation of APC during acute hyperglycemia was also linked to regulation of miR-21; i.e., overexpression of miR-21 in cells exposed to high glucose restored APC via the Akt/GSK3β link and increased cell survival.

### Mitochondrial DNA

Mitochondria have their own genome that comprises only a small portion of the total eukaryotic cell genome. The mitochondrial DNA (mDNA) encodes 13 mitochondrial proteins and the mitochondrial rRNAs and tRNAs needed for translation (Kirby and Thorburn, 2008). Unlike the nuclear DNA, mDNA is not protected by histones and is therefore susceptible to damage by oxidative stress (Camara et al., 2010). A decrease in mDNA is a biological maker of myocardial damage, as in cardiac hypertrophy, that progresses to heart failure (Karamanlidis et al., 2011). A recent study (Muravyeva et al., 2014) examined if mDNA modulates APC and cardiac susceptibility to IR injury by using two strains of diabetic rats following exposure to isoflurane. The study proposed that differences in the mitochondrial genome modulate isoflurane-induced generation of ROS that translates into a differential susceptibility to APC; this suggested a potentially important role of mDNA in regulating cardioprotection in APC via modulation of ROS production.

## Conclusions and future directions

Improvement in the clinical management of ischemic heart disease remains elusive despite the discovery of many molecular and cellular mechanisms that may be valuable targets to treat against IR injury. The importance of mitochondrial bioenergetics and function in contributing not only to cardiac injury but also to reducing cardiac injury is now well recognized. But there remains a lack of clear understanding of the mitochondrial-cytosolic mechanisms that might lead to more targeted intervention. Hence, we need to identify new targets that could uncover the mechanisms of dysfunction associated with IR injury. With a better understanding of mitochondrial targets as hubs for controlling metabolism and cellular redox signaling pathways that elicit protection, we could better develop novel therapeutic drugs for clinical trials to protect against IR injury. Because VA are lipophilic agents with multi-targeted actions that, together, confer cardioprotection, they give us valuable clues into which potential sites to investigate; these clues may be especially useful to selectively and reversibly target mitochondria to reduce IR injury. Unfortunately, as summarized in this review, there is contradictory evidence with respect to the potentially large number of pathways by which VA might protect the heart. Nonetheless, there are molecules with characteristics of a VA but without anesthetic properties. These could be developed as cardioprotective drugs while obviating the need for inducing anesthesia.

### Conflict of interest statement

The authors declare that the research was conducted in the absence of any commercial or financial relationships that could be construed as a potential conflict of interest.

## Abbreviations

VA: volatile anesthetics
IR: ischemia and reperfusion
ETC: electron transport chain
ROS: reactive oxygen species
ΔΨ_m_: mitochondrial membrane potential
MI: myocardial ischemia
CAD: coronary artery disease
OxPhos: oxidative phosphorylation
IPC: ischemic pre-conditioning
PKB: protein kinase B
PKC: protein kinase C
PKA: protein kinase A
ERK: extracellular regulated kinases
APC: anesthetic-preconditioning
RISK: reperfusion injury salvage kinase
CaMK: calcium/calmodulin-dependent protein kinases
GSK: glycogen synthase kinase
RNS: reactive nitrogen species
mK_ATP_: mitochondrial ATP sensitive K^+^-channel
mBK_Ca_: mitochondrial big Ca^2+^ sensitive K^+^ channel
mPTP: kilodalton (kDa), mitochondrial permeability transition pore
mNCE: mitochondrial Na^+^-Ca^2+^ exchanger
OMM: outer mitochondrial membrane
IMM: inner mitochondrial membrane
VDAC: voltage-dependent anion channel
PTMs: post-translational modifications
ATP: adenosine triphosphate
IP3: inositol triphosphate receptor
ANT: adenosine nucleotide transport
TCA: tricarboxylic acid
ADP: adenosine triphosphate
NHE: sodium hydrogen exchanger
NCE: sodium calcium exchanger
5-HD: 5-hydroxydecanoic acid
mCU: mitochondrial calcium uniporter
CyP-D: cyclophilin D
PPIase: peptidylprolyl cis-trans isomerase
CSA: cyclosporin A
eNOS: endothelial nitric oxide synthase
APoC: anesthetic-post-conditioning
GPR: G protein coupled receptor
HSP: heat shock protein
ALDH2: aldehyde dehydrogenase 2
4-HNE: 4-Hydroxynonenal
Q^·^: ubisemiquinone
UCPs: uncoupling proteins
NF-κB: nuclear factor–κB
O-GlcNAc: O-linked-β-N-acetylglucosamine
miRNA: micro RNA
mDNA: mitochondrial DNA.

## References

[B1] Agarwal B. Camara A. K. Stowe D. F. Bosnjak Z. J. Dash R. K. (2012). Enhanced charge-independent mitochondrial free Ca^2+^ and attenuated ADP-induced NADH oxidation by isoflurane: implications for cardioprotection. Biochim. Biophys. Acta 1817, 453–465 10.1016/j.bbabio.2011.11.011 22155157 PMC3269543

[B2] Agarwal B. Dash R. K. Stowe D. F. Bosnjak Z. J. Camara A. K. (2014). Isoflurane modulates cardiac mitochondrial bioenergetics by selectively attenuating respiratory complexes. Biochim. Biophys. Acta 1837, 354–365 10.1016/j.bbabio.2013.11.006 24355434 PMC4084852

[B3] Aldakkak M. Stowe D. F. Chen Q. Lesnefsky E. J. Camara A. K. (2008). Inhibited mitochondrial respiration by amobarbital during cardiac ischaemia improves redox state and reduces matrix Ca^2+^ overload and ROS release. Cardiovasc. Res. 77, 406–415 10.1016/j.cardiores.2007.08.008 17900548

[B4] An J. Varadarajan S. G. Novalija E. Stowe D. F. (2001). Ischemic and anesthetic preconditioning reduces cytosolic [Ca^2+^] and improves Ca^2+^ responses in intact hearts. Am. J. Physiol. Heart Circ. Physiol. 281, H1508–H1523 11557539 10.1152/ajpheart.2001.281.4.H1508

[B5] Arrell D. K. Elliott S. T. Kane L. A. Guo Y. Ko Y. H. Pedersen P. L. (2006). Proteomic analysis of pharmacological preconditioning: novel protein targets converge to mitochondrial metabolism pathways. Circ. Res. 99, 706–714 10.1161/01.RES.0000243995.74395.f8 16946135

[B6] Baines C. P. Song C. X. Zheng Y. T. Wang G. W. Zhang J. Wang O. L. (2003). Protein kinase Cepsilon interacts with and inhibits the permeability transition pore in cardiac mitochondria. Circ. Res. 92, 873–880 10.1161/01.RES.0000069215.36389.8D 12663490 PMC3691672

[B7] Belhomme D. Peynet J. Louzy M. Launay J. M. Kitakaze M. Menasche P. (1999). Evidence for preconditioning by isoflurane in coronary artery bypass graft surgery. Circulation 100, II340–II344 10.1161/01.CIR.100.suppl_2.II-340 10567326

[B8] Bernardi P. (2013). The mitochondrial permeability transition pore: a mystery solved? Front. Physiol. 4:95 10.3389/fphys.2013.00095 23675351 PMC3650560

[B9] Bertaccini E. J. Trudell J. R. Franks N. P. (2007). The common chemical motifs within anesthetic binding sites. Anesth. Analg. 104, 318–324 10.1213/01.ane.0000253029.67331.8d 17242087

[B10] Bienengraeber M. Pellitteri-Hahn M. Hirata N. Baye T. M. Bosnjak Z. J. Olivier M. (2013). Quantitative characterization of changes in the cardiac mitochondrial proteome during anesthetic preconditioning and ischemia. Physiol. Genomics 45, 163–170 10.1152/physiolgenomics.00117.2012 23300156 PMC3615574

[B11] Boelens A. D. Pradhan R. K. Blomeyer C. A. Camara A. K. Dash R. K. Stowe D. F. (2013). Extra-matrix Mg^2+^ limits Ca^2+^ uptake and modulates Ca^2+^ uptake-independent respiration and redox state in cardiac isolated mitochondria. J. Bioenerg. Biomembr. 45, 203–218 10.1007/s10863-013-9500-5 23456198 PMC3670670

[B12] Bopassa J. C. Eghbali M. Toro L. Stefani E. (2010). A novel estrogen receptor GPER inhibits mitochondria permeability transition pore opening and protects the heart against ischemia-reperfusion injury. Am. J. Physiol. Heart Circ. Physiol. 298, H16–H23 10.1152/ajpheart.00588.2009 19880667 PMC2806134

[B13] Bosnjak Z. J. Kampine J. P. (1986). Effects of halothane on transmembrane potentials, Ca^2+^ transients, and papillary muscle tension in the cat. Am. J. Physiol. 251, H374–381 3740291 10.1152/ajpheart.1986.251.2.H374

[B14] Bouwman R. A. Salic K. Padding F. G. Eringa E. C. van Beek-Harmsen B. J. Matsuda T. (2006). Cardioprotection via activation of protein kinase C-delta depends on modulation of the reverse mode of the Na^+^/Ca^2+^ exchanger. Circulation 114, I226–I232 10.1161/CIRCULATIONAHA.105.000570 16820577

[B15] Brookes P. S. Yoon Y. Robotham J. L. Anders M. W. Sheu S. S. (2004). Calcium, ATP, and ROS: a mitochondrial love-hate triangle. Am. J. Physiol. Cell Physiol. 287, C817–C833 10.1152/ajpcell.00139.2004 15355853

[B16] Brookes P. S. Zhang J. Dai L. Zhou F. Parks D. A. Darley-Usmar V. M. (2001). Increased sensitivity of mitochondrial respiration to inhibition by nitric oxide in cardiac hypertrophy. J. Mol. Cell. Cardiol. 33, 69–82 10.1006/jmcc.2000.1276 11133224

[B17] Budas G. R. Churchill E. N. Disatnik M. H. Sun L. Mochly-Rosen D. (2010). Mitochondrial import of PKCepsilon is mediated by HSP90: a role in cardioprotection from ischaemia and reperfusion injury. Cardiovasc. Res. 88, 83–92 10.1093/cvr/cvq154 20558438 PMC2936125

[B18] Burwell L. S. Nadtochiy S. M. Tompkins A. J. Young S. Brookes P. S. (2006). Direct evidence for S-nitrosation of mitochondrial complex I. Biochem. J. 394, 627–634 10.1042/BJ20051435 16371007 PMC1383712

[B19] Camara A. K. Bienengraeber M. Stowe D. F. (2011). Mitochondrial approaches to protect against cardiac ischemia and reperfusion injury. Front. Physiol. 2:13 10.3389/fphys.2011.00013 21559063 PMC3082167

[B20] Camara A. K. Lesnefsky E. J. Stowe D. F. (2010). Potential therapeutic benefits of strategies directed to mitochondria. Antioxid. Redox Signal. 13, 279–347 10.1089/ars.2009.2788 20001744 PMC2936955

[B21] Casanovas A. M. Malmary Nebot M. F. Courriere P. Oustrin J. (1983). Inhibition of cytochrome oxidase activity by local anaesthetics. Biochem. Pharmacol. 32, 2715–2719 10.1016/0006-2952(83)90081-3 6313007

[B22] Chakrabarti A. K. Feeney K. Abueg C. Brown D. A. Czyz E. Tendera M. (2013). Rationale and design of the EMBRACE STEMI study: a phase 2a, randomized, double-blind, placebo-controlled trial to evaluate the safety, tolerability and efficacy of intravenous Bendavia on reperfusion injury in patients treated with standard therapy including primary percutaneous coronary intervention and stenting for ST-segment elevation myocardial infarction. Am. Heart J. 165, 509–514.e7 10.1016/j.ahj.2012.12.008 23537966

[B23] Champattanachai V. Marchase R. B. Chatham J. C. (2008). Glucosamine protects neonatal cardiomyocytes from ischemia-reperfusion injury via increased protein O-GlcNAc and increased mitochondrial Bcl-2. Am. J. Physiol. Cell Physiol. 294, C1509–C1520 10.1152/ajpcell.00456.2007 18367586 PMC2800950

[B24] Chen K. Rajewsky N. (2007). The evolution of gene regulation by transcription factors and microRNAs. Nat. Rev. Genet. 8, 93–103 10.1038/nrg1990 17230196

[B25] Chen Q. Camara A. K. An J. Novalija E. Riess M. L. Stowe D. F. (2002). Sevoflurane preconditioning before moderate hypothermic ischemia protects against cytosolic [Ca^2+^] loading and myocardial damage in part via mitochondrial K_ATP_ channels. Anesthesiology 97, 912–920 10.1097/00000542-200210000-00025 12357159

[B26] Chen Q. Camara A. K. Stowe D. F. Hoppel C. L. Lesnefsky E. J. (2007). Modulation of electron transport protects cardiac mitochondria and decreases myocardial injury during ischemia and reperfusion. Am. J. Physiol. Cell Physiol. 292, C137–C147 10.1152/ajpcell.00270.2006 16971498

[B27] Chen Q. Vazquez E. J. Moghaddas S. Hoppel C. L. Lesnefsky E. J. (2003). Production of reactive oxygen species by mitochondria: central role of complex III. J. Biol. Chem. 278, 36027–36031 10.1074/jbc.M304854200 12840017

[B28] Chen Z. Chua C. C. Ho Y. S. Hamdy R. C. Chua B. H. (2001). Overexpression of Bcl-2 attenuates apoptosis and protects against myocardial I/R injury in transgenic mice. Am. J. Physiol. Heart Circ. Physiol. 280, H2313–H2320 11299236 10.1152/ajpheart.2001.280.5.H2313

[B29] Cheng Y. Tan N. Yang J. Liu X. Cao X. He P. (2010a). A translational study of circulating cell-free microRNA-1 in acute myocardial infarction. Clin. Sci. 119, 87–95 10.1042/CS20090645 20218970 PMC3593815

[B30] Cheng Y. Zhu P. Yang J. Liu X. Dong S. Wang X. (2010b). Ischaemic preconditioning-regulated miR-21 protects heart against ischaemia/reperfusion injury via anti-apoptosis through its target PDCD4. Cardiovasc. Res. 87, 431–439 10.1093/cvr/cvq082 20219857 PMC2904662

[B31] Chiari P. C. Bienengraeber M. W. Pagel P. S. Krolikowski J. G. Kersten J. R. Warltier D. C. (2005a). Isoflurane protects against myocardial infarction during early reperfusion by activation of phosphatidylinositol-3-kinase signal transduction: evidence for anesthetic-induced postconditioning in rabbits. Anesthesiology 102, 102–109 10.1097/00000542-200501000-00018 15618793

[B32] Chiari P. C. Bienengraeber M. W. Weihrauch D. Krolikowski J. G. Kersten J. R. Warltier D. C. (2005b). Role of endothelial nitric oxide synthase as a trigger and mediator of isoflurane-induced delayed preconditioning in rabbit myocardium. Anesthesiology 103, 74–83 10.1097/00000542-200507000-00014 15983459

[B33] Clarke S. J. McStay G. P. Halestrap A. P. (2002). Sanglifehrin A acts as a potent inhibitor of the mitochondrial permeability transition and reperfusion injury of the heart by binding to cyclophilin-D at a different site from cyclosporin A. J. Biol. Chem. 277, 34793–34799 10.1074/jbc.M202191200 12095984

[B34] Craigen W. J. Graham B. H. (2008). Genetic strategies for dissecting mammalian and Drosophila voltage-dependent anion channel functions. J. Bioenerg. Biomembr. 40, 207–212 10.1007/s10863-008-9146-x 18622693 PMC4822497

[B35] Crompton M. Virji S. Ward J. M. (1998). Cyclophilin-D binds strongly to complexes of the voltage-dependent anion channel and the adenine nucleotide translocase to form the permeability transition pore. Eur. J. Biochem. 258, 729–735 10.1046/j.1432-1327.1998.2580729.x 9874241

[B36] Das S. Steenbergen C. Murphy E. (2012). Does the voltage dependent anion channel modulate cardiac ischemia-reperfusion injury? Biochim. Biophys. Acta 1818, 1451–1456 10.1016/j.bbamem.2011.11.008 22100866 PMC3382059

[B37] Das S. Wong R. Rajapakse N. Murphy E. Steenbergen C. (2008). Glycogen synthase kinase 3 inhibition slows mitochondrial adenine nucleotide transport and regulates voltage-dependent anion channel phosphorylation. Circ. Res. 103, 983–991 10.1161/CIRCRESAHA.108.178970 18802025 PMC2661871

[B38] De Hert S. G. ten Broecke P. W. Mertens E. Van Sommeren E. W. De Blier I. G. Stockman B. A. (2002). Sevoflurane but not propofol preserves myocardial function in coronary surgery patients. Anesthesiology 97, 42–49 10.1097/00000542-200207000-00007 12131102

[B39] De Hert S. G. Turani F. Mathur S. Stowe D. F. (2005). Cardioprotection with volatile anesthetics: mechanisms and clinical implications. Anesth. Analg. 100, 1584–1593 10.1213/01.ANE.0000153483.61170.0C 15920178

[B40] De Stefani D. Bononi A. Romagnoli A. Messina A. De Pinto V. Pinton P. (2012). VDAC1 selectively transfers apoptotic Ca^2+^ signals to mitochondria. Cell Death Differ. 19, 267–273 10.1038/cdd.2011.92 21720385 PMC3263501

[B41] Dong S. Cheng Y. Yang J. Li J. Liu X. Wang X. (2009). MicroRNA expression signature and the role of microRNA-21 in the early phase of acute myocardial infarction. J. Biol. Chem. 284, 29514–29525 10.1074/jbc.M109.027896 19706597 PMC2785585

[B42] Duchen M. R. (2004). Mitochondria in health and disease: perspectives on a new mitochondrial biology. Mol. Aspects Med. 25, 365–451 10.1016/j.mam.2004.03.001 15302203

[B43] Eckenhoff R. G. Johansson J. S. (1997). Molecular interactions between inhaled anesthetics and proteins. Pharmacol. Rev. 49, 343–367 9443162

[B44] Facundo H. T. Carreira R. S. de Paula J. G. Santos C. C. Ferranti R. Laurindo F. R. (2006a). Ischemic preconditioning requires increases in reactive oxygen release independent of mitochondrial K^+^ channel activity. Free Radic. Biol. Med. 40, 469–479 10.1016/j.freeradbiomed.2005.08.041 16443162

[B45] Facundo H. T. Fornazari M. Kowaltowski A. J. (2006b). Tissue protection mediated by mitochondrial K^+^ channels. Biochim. Biophys. Acta 1762, 202–212 10.1016/j.bbadis.2005.06.003 16026967

[B46] Feng J. Zhu M. Schaub M. C. Gehrig P. Roschitzki B. Lucchinetti E. (2008). Phosphoproteome analysis of isoflurane-protected heart mitochondria: phosphorylation of adenine nucleotide translocator-1 on Tyr194 regulates mitochondrial function. Cardiovasc. Res. 80, 20–29 10.1093/cvr/cvn161 18558627

[B47] Ferrari R. (1996). The role of mitochondria in ischemic heart disease. J. Cardiovasc. Pharmacol. 28(Suppl. 1), S1–S10 8891865 10.1097/00005344-199600003-00002

[B48] Fleisher L. A. Beckman J. A. Brown K. A. Calkins H. Chaikof E. L. Fleischmann K. E. (2007). ACC/AHA 2007 guidelines on perioperative cardiovascular evaluation and care for noncardiac surgery: executive summary: a report of the American college of cardiology/American heart association task force on practice guidelines (writing committee to revise the 2002 guidelines on perioperative cardiovascular evaluation for noncardiac surgery) developed in collaboration with the American society of echocardiography, American society of nuclear cardiology, heart rhythm society, society of cardiovascular anesthesiologists, society for cardiovascular angiography and interventions, society for vascular medicine and biology, and society for vascular surgery. J. Am. Coll. Cardiol. 50, 1707–1732 10.1016/j.jacc.2007.09.001 17950159

[B49] Foster D. B. Ho A. S. Rucker J. Garlid A. O. Chen L. Sidor A. (2012). Mitochondrial ROMK channel is a molecular component of mitoK_ATP_. Circ. Res. 111, 446–454 10.1161/CIRCRESAHA.112.266445 22811560 PMC3560389

[B50] Foster D. B. Van Eyk J. E. Marban E. O'Rourke B. (2009). Redox signaling and protein phosphorylation in mitochondria: progress and prospects. J. Bioenerg. Biomembr. 41, 159–168 10.1007/s10863-009-9217-7 19440831 PMC2921908

[B51] Freedman B. M. Hamm D. P. Everson C. T. Wechsler A. S. Christian C. M. 2nd. (1985). Enflurane enhances postischemic functional recovery in the isolated rat heart. Anesthesiology 62, 29–33 10.1097/00000542-198501000-00006 3966666

[B52] Garesse R. Vallejo C. G. (2001). Animal mitochondrial biogenesis and function: a regulatory cross-talk between two genomes. Gene 263, 1–16 10.1016/S0378-1119(00)00582-5 11223238

[B53] Ge Z. D. Pravdic D. Bienengraeber M. Pratt P. F. Jr. Auchampach J. A. Gross G. J. (2010). Isoflurane postconditioning protects against reperfusion injury by preventing mitochondrial permeability transition by an endothelial nitric oxide synthase-dependent mechanism. Anesthesiology 112, 73–85 10.1097/ALN.0b013e3181c4a607 19996950 PMC4374483

[B54] Gerstenblith G. (2004). Derangements in cardiac metabolism in the ischemic state and consequences of reperfusion. Adv. Stud. Med. 4, 464–471 7748481

[B55] Giorgio V. von Stockum S. Antoniel M. Fabbro A. Fogolari F. Forte M. (2013). Dimers of mitochondrial ATP synthase form the permeability transition pore. Proc. Natl. Acad. Sci. U.S.A. 110, 5887–5892 10.1073/pnas.1217823110 23530243 PMC3625323

[B56] Gross E. R. Hsu A. K. Gross G. J. (2008). Delayed cardioprotection afforded by the glycogen synthase kinase 3 inhibitor SB-216763 occurs via a K_ATP_- and MPTP-dependent mechanism at reperfusion. Am. J. Physiol. Heart Circ. Physiol. 294, H1497–H1500 10.1152/ajpheart.01381.2007 18223186

[B57] Gross G. J. Fryer R. M. (1999). Sarcolemmal versus mitochondrial ATP-sensitive K^+^ channels and myocardial preconditioning. Circ. Res. 84, 973–979 10.1161/01.RES.84.9.973 10325234

[B58] Halestrap A. P. (2010). A pore way to die: the role of mitochondria in reperfusion injury and cardioprotection. Biochem. Soc. Trans. 38, 841–860 10.1042/BST0380841 20658967

[B59] Hanley P. J. Mickel M. Loffler M. Brandt U. Daut J. (2002). K_ATP_ channel-independent targets of diazoxide and 5-hydroxydecanoate in the heart. J. Physiol. 542, 735–741 10.1113/jphysiol.2002.023960 12154175 PMC2290447

[B60] Hausenloy D. J. Maddock H. L. Baxter G. F. Yellon D. M. (2002). Inhibiting mitochondrial permeability transition pore opening: a new paradigm for myocardial preconditioning? Cardiovasc. Res. 55, 534–543 10.1016/S0008-6363(02)00455-8 12160950

[B61] He B. Xiao J. Ren A. J. Zhang Y. F. Zhang H. Chen M. (2011). Role of miR-1 and miR-133a in myocardial ischemic postconditioning. J. Biomed. Sci. 18:22 10.1186/1423-0127-18-22 21406115 PMC3066105

[B62] Hemmings H. C. Jr. (2010). Molecular targets of general anesthetics in the nervous system, in Supressing the Mind: Anesthetics Modulation of Memory and Consciousness, Chapter 2, eds Hudetz A. Pearce R. (New York, NY: Humana Press Springer), 11–31

[B63] Hirata N. Shim Y. H. Pravdic D. Lohr N. L. Pratt P. F. Jr. Weihrauch D. (2011). Isoflurane differentially modulates mitochondrial reactive oxygen species production via forward versus reverse electron transport flow: implications for preconditioning. Anesthesiology 115, 531–540 10.1097/ALN.0b013e31822a2316 21862887 PMC3337729

[B64] Hirose K. Tsutsumi Y. M. Tsutsumi R. Shono M. Katayama E. Kinoshita M. (2011). Role of the O-linked beta-N-acetylglucosamine in the cardioprotection induced by isoflurane. Anesthesiology 115, 955–962 10.1097/ALN.0b013e31822fcede 21876430

[B65] Hodge T. Colombini M. (1997). Regulation of metabolite flux through voltage-gating of VDAC channels. J. Membr. Biol. 157, 271–279 10.1007/s002329900235 9178614

[B66] Hu Z. Y. Liu J. (2009). Mechanism of cardiac preconditioning with volatile anaesthetics. Anaesth. Intensive Care 37, 532–538 19681408 10.1177/0310057X0903700402

[B67] Imahashi K. Schneider M. D. Steenbergen C. Murphy E. (2004). Transgenic expression of Bcl-2 modulates energy metabolism, prevents cytosolic acidification during ischemia, and reduces ischemia/reperfusion injury. Circ. Res. 95, 734–741 10.1161/01.RES.0000143898.67182.4c 15345651

[B68] Jamnicki-Abegg M. Weihrauch D. Pagel P. S. Kersten J. R. Bosnjak Z. J. Warltier D. C. (2005). Isoflurane inhibits cardiac myocyte apoptosis during oxidative and inflammatory stress by activating Akt and enhancing Bcl-2 expression. Anesthesiology 103, 1006–1014 10.1097/00000542-200511000-00015 16249675

[B69] Javadov S. A. Clarke S. Das M. Griffiths E. J. Lim K. H. Halestrap A. P. (2003). Ischaemic preconditioning inhibits opening of mitochondrial permeability transition pores in the reperfused rat heart. J. Physiol. 549, 513–524 10.1113/jphysiol.2003.034231 12692185 PMC2342939

[B70] Javadov S. Karmazyn M. Escobales N. (2009). Mitochondrial permeability transition pore opening as a promising therapeutic target in cardiac diseases. J. Pharmacol. Exp. Ther. 330, 670–678 10.1124/jpet.109.153213 19509316

[B71] Jenkins A. Greenblatt E. P. Faulkner H. J. Bertaccini E. Light A. Lin A. (2001). Evidence for a common binding cavity for three general anesthetics within the GABAA receptor. J. Neurosci. 21, RC136 11245705 10.1523/JNEUROSCI.21-06-j0002.2001 PMC6762625

[B72] Jiang M. T. Nakae Y. Ljubkovic M. Kwok W. M. Stowe D. F. Bosnjak Z. J. (2007). Isoflurane activates human cardiac mitochondrial adenosine triphosphate-sensitive K+ channels reconstituted in lipid bilayers. Anesth. Analg. 105, 926–932 10.1213/01.ane.0000278640.81206.92 17898367

[B73] Jones D. A. Andiapen M. Van-Eijl T. J. Webb A. J. Antoniou S. Schilling R. J. (2013). The safety and efficacy of intracoronary nitrite infusion during acute myocardial infarction (NITRITE-AMI): study protocol of a randomised controlled trial. BMJ Open 3, 1–8 10.1136/bmjopen-2013-002813 23550096 PMC3641434

[B74] Jones S. P. Zachara N. E. Ngoh G. A. Hill B. G. Teshima Y. Bhatnagar A. (2008). Cardioprotection by N-acetylglucosamine linkage to cellular proteins. Circulation 117, 1172–1182 10.1161/CIRCULATIONAHA.107.730515 18285568

[B75] Jovic M. Stancic A. Nenadic D. Cekic O. Nezic D. Milojevic P. (2012). Mitochondrial molecular basis of sevoflurane and propofol cardioprotection in patients undergoing aortic valve replacement with cardiopulmonary bypass. Cell. Physiol. Biochem. 29, 131–142 10.1159/000337594 22415082

[B76] Juhaszova M. Zorov D. B. Kim S. H. Pepe S. Fu Q. Fishbein K. W. (2004). Glycogen synthase kinase-3beta mediates convergence of protection signaling to inhibit the mitochondrial permeability transition pore. J. Clin. Invest. 113, 1535–1549 10.1172/JCI19906 15173880 PMC419483

[B77] Julier K. da Silva R. Garcia C. Bestmann L. Frascarolo P. Zollinger A. (2003). Preconditioning by sevoflurane decreases biochemical markers for myocardial and renal dysfunction in coronary artery bypass graft surgery: a double-blinded, placebo-controlled, multicenter study. Anesthesiology 98, 1315–1327 10.1097/00000542-200306000-00004 12766638

[B78] Kalenka A. Maurer M. H. Feldmann R. E. Kuschinsky W. Waschke K. F. (2006). Volatile anesthetics evoke prolonged changes in the proteome of the left ventricule myocardium: defining a molecular basis of cardioprotection? Acta Anaesthesiol. Scand. 50, 414–427 10.1111/j.1399-6576.2006.00984.x 16548853

[B79] Karamanlidis G. Bautista-Hernandez V. Fynn-Thompson F. Del Nido P. Tian R. (2011). Impaired mitochondrial biogenesis precedes heart failure in right ventricular hypertrophy in congenital heart disease. Circ. Heart Fail. 4, 707–713 10.1161/CIRCHEARTFAILURE.111.961474 21840936 PMC3218261

[B80] Kersten J. R. Schmeling T. J. Hettrick D. A. Pagel P. S. Gross G. J. Warltier D. C. (1996). Mechanism of myocardial protection by isoflurane. Role of adenosine triphosphate-regulated potassium (KATP) channels. Anesthesiology 85, 794–807 10.1097/00000542-199610000-00015 8873550

[B81] Kersten J. R. Schmeling T. J. Pagel P. S. Gross G. J. Warltier D. C. (1997). Isoflurane mimics ischemic preconditioning via activation of K_ATP_ channels: reduction of myocardial infarct size with an acute memory phase. Anesthesiology 87, 361–370 10.1097/00000542-199708000-00024 9286901

[B82] Kevin L. G. Novalija E. Riess M. L. Camara A. K. Rhodes S. S. Stowe D. F. (2003). Sevoflurane exposure generates superoxide but leads to decreased superoxide during ischemia and reperfusion in isolated hearts. Anesth. Analg. 96, 949–955 10.1213/01.ANE.0000052515.25465.35 12651639

[B83] Kevin L. G. Novalija E. Stowe D. F. (2005). Reactive oxygen species as mediators of cardiac injury and protection: the relevance to anesthesia practice. Anesth. Analg. 101, 1275–1287 10.1213/01.ANE.0000180999.81013.D0 16243980

[B84] Kirby D. M. Thorburn D. R. (2008). Approaches to finding the molecular basis of mitochondrial oxidative phosphorylation disorders. Twin Res. Hum. Genet. 11, 395–411 10.1375/twin.11.4.395 18637740

[B85] Koltchine V. V. Finn S. E. Jenkins A. Nikolaeva N. Lin A. Harrison N. L. (1999). Agonist gating and isoflurane potentiation in the human gamma-aminobutyric acid type A receptor determined by the volume of a second transmembrane domain residue. Mol. Pharmacol. 56, 1087–1093 10531417 10.1124/mol.56.5.1087

[B86] Krolikowski J. G. Bienengraeber M. Weihrauch D. Warltier D. C. Kersten J. R. Pagel P. S. (2005). Inhibition of mitochondrial permeability transition enhances isoflurane-induced cardioprotection during early reperfusion: the role of mitochondrial K_ATP_ channels. Anesth. Analg. 101, 1590–1596 10.1213/01.ANE.0000181288.13549.28 16301224

[B87] Krolikowski J. G. Weihrauch D. Bienengraeber M. Kersten J. R. Warltier D. C. Pagel P. S. (2006). Role of Erk1/2, p70s6K, and eNOS in isoflurane-induced cardioprotection during early reperfusion *in vivo*. Can. J. Anaesth. 53, 174–182 10.1007/BF03021824 16434759

[B88] Lang X. E. Wang X. Zhang K. R. Lv J. Y. Jin J. H. Li Q. S. (2013). Isoflurane preconditioning confers cardioprotection by activation of ALDH2. PLoS ONE 8:e52469 10.1371/journal.pone.0052469 23468836 PMC3585331

[B89] Lemasters J. J. Holmuhamedov E. (2006). Voltage-dependent anion channel (VDAC) as mitochondrial governator–thinking outside the box. Biochim. Biophys. Acta 1762, 181–190 10.1016/j.bbadis.2005.10.006 16307870

[B90] Lim K. H. Javadov S. A. Das M. Clarke S. J. Suleiman M. S. Halestrap A. P. (2002). The effects of ischaemic preconditioning, diazoxide and 5-hydroxydecanoate on rat heart mitochondrial volume and respiration. J. Physiol. 545, 961–974 10.1113/jphysiol.2002.031484 12482899 PMC2290722

[B91] Liu B. Tewari A. K. Zhang L. Green-Church K. B. Zweier J. L. Chen Y. R. (2009). Proteomic analysis of protein tyrosine nitration after ischemia reperfusion injury: mitochondria as the major target. Biochim. Biophys. Acta 1794, 476–485 10.1016/j.bbapap.2008.12.008 19150419 PMC2637933

[B92] Liu H. Wang L. Eaton M. Schaefer S. (2005). Sevoflurane preconditioning limits intracellular/mitochondrial Ca^2+^ in ischemic newborn myocardium. Anesth. Analg. 101, 349–355 10.1213/01.ANE.0000154197.24763.EC 16037142

[B93] Ljubkovic M. Mio Y. Marinovic J. Stadnicka A. Warltier D. C. Bosnjak Z. J. (2007). Isoflurane preconditioning uncouples mitochondria and protects against hypoxia-reoxygenation. Am. J. Physiol. Cell Physiol. 292, C1583–C1590 10.1152/ajpcell.00221.2006 17215328

[B94] Ludwig L. M. Tanaka K. Eells J. T. Weihrauch D. Pagel P. S. Kersten J. R. (2004a). Preconditioning by isoflurane is mediated by reactive oxygen species generated from mitochondrial electron transport chain complex III. Anesth. Analg. 99, 1308–1315 10.1213/01.ANE.0000134804.09484.5D 15502022

[B95] Ludwig L. M. Weihrauch D. Kersten J. R. Pagel P. S. Warltier D. C. (2004b). Protein kinase C translocation and Src protein tyrosine kinase activation mediate isoflurane-induced preconditioning *in vivo*: potential downstream targets of mitochondrial adenosine triphosphate-sensitive potassium channels and reactive oxygen species. Anesthesiology 100, 532–539 10.1097/00000542-200403000-00011 15108965

[B96] Marinovic J. Bosnjak Z. J. Stadnicka A. (2006). Distinct roles for sarcolemmal and mitochondrial adenosine triphosphate-sensitive potassium channels in isoflurane-induced protection against oxidative stress. Anesthesiology 105, 98–104 10.1097/00000542-200607000-00018 16810000

[B97] Mewton N. Croisille P. Gahide G. Rioufol G. Bonnefoy E. Sanchez I. (2010). Effect of cyclosporine on left ventricular remodeling after reperfused myocardial infarction. J. Am. Coll. Cardiol. 55, 1200–1205 10.1016/j.jacc.2009.10.052 20298926

[B98] Mio Y. Uezono S. Kitahata H. (2014). Anesthetic cardioprotection in relation to mitochondria: basic science. Curr. Pharm. Des. [Epub ahead of print]. 10.2174/1381612820666140204110101 24502577

[B99] Muravyeva M. Baotic I. Bienengraeber M. Lazar J. Bosnjak Z. J. Sedlic F. (2014). Cardioprotection during diabetes: the role of mitochondrial DNA. Anesthesiology 120, 870–879 10.1097/ALN.0000000000000107 24346177 PMC3975667

[B100] Murphy E. Steenbergen C. (2008a). Ion transport and energetics during cell death and protection. Physiology (Bethesda) 23, 115–123 10.1152/physiol.00044.2007 18400694 PMC2872775

[B101] Murphy E. Steenbergen C. (2008b). Mechanisms underlying acute protection from cardiac ischemia-reperfusion injury. Physiol. Rev. 88, 581–609 10.1152/physrev.00024.2007 18391174 PMC3199571

[B102] Murphy M. P. Echtay K. S. Blaikie F. H. Asin-Cayuela J. Cocheme H. M. Green K. (2003). Superoxide activates uncoupling proteins by generating carbon-centered radicals and initiating lipid peroxidation: studies using a mitochondria-targeted spin trap derived from alpha-phenyl-N-tert-butylnitrone. J. Biol. Chem. 278, 48534–48545 10.1074/jbc.M308529200 12972420

[B103] Murray J. Taylor S. W. Zhang B. Ghosh S. S. Capaldi R. A. (2003). Oxidative damage to mitochondrial complex I due to peroxynitrite: identification of reactive tyrosines by mass spectrometry. J. Biol. Chem. 278, 37223–37230 10.1074/jbc.M305694200 12857734

[B104] Murry C. E. Jennings R. B. Reimer K. A. (1986). Preconditioning with ischemia: a delay of lethal cell injury in ischemic myocardium. Circulation 74, 1124–1136 10.1161/01.CIR.74.5.1124 3769170

[B105] Nadtochiy S. M. Tompkins A. J. Brookes P. S. (2006). Different mechanisms of mitochondrial proton leak in ischaemia/reperfusion injury and preconditioning: implications for pathology and cardioprotection. Biochem. J. 395, 611–618 10.1042/BJ20051927 16436046 PMC1462692

[B106] Neumann D. Buckers J. Kastrup L. Hell S. W. Jakobs S. (2010). Two-color STED microscopy reveals different degrees of colocalization between hexokinase-I and the three human VDAC isoforms. PMC Biophys. 3:4 10.1186/1757-5036-3-4 20205711 PMC2838807

[B107] Nickel A. Kohlhaas M. Maack C. (2014). Mitochondrial reactive oxygen species production and elimination. J. Mol. Cell. Cardiol. 73, 26–33 10.1016/j.yjmcc.2014.03.011 24657720

[B108] Nicolli A. Basso E. Petronilli V. Wenger R. M. Bernardi P. (1996). Interactions of cyclophilin with the mitochondrial inner membrane and regulation of the permeability transition pore, and cyclosporin A-sensitive channel. J. Biol. Chem. 271, 2185–2192 10.1074/jbc.271.4.2185 8567677

[B109] Nishihara M. Miura T. Miki T. Sakamoto J. Tanno M. Kobayashi H. (2006). Erythropoietin affords additional cardioprotection to preconditioned hearts by enhanced phosphorylation of glycogen synthase kinase-3 beta. Am. J. Physiol. Heart Circ. Physiol. 291, H748–H755 10.1152/ajpheart.00837.2005 16565311

[B110] Nishihara M. Miura T. Miki T. Tanno M. Yano T. Naitoh K. (2007). Modulation of the mitochondrial permeability transition pore complex in GSK-3beta-mediated myocardial protection. J. Mol. Cell. Cardiol. 43, 564–570 10.1016/j.yjmcc.2007.08.010 17931653

[B111] Novalija E. Fujita S. Kampine J. P. Stowe D. F. (1999). Sevoflurane mimics ischemic preconditioning effects on coronary flow and nitric oxide release in isolated hearts. Anesthesiology 91, 701–712 10.1097/00000542-199909000-00023 10485782

[B112] Novalija E. Kevin L. G. Camara A. K. Bosnjak Z. J. Kampine J. P. Stowe D. F. (2003). Reactive oxygen species precede the epsilon isoform of protein kinase C in the anesthetic preconditioning signaling cascade. Anesthesiology 99, 421–428 10.1097/00000542-200308000-00024 12883415

[B113] Novalija E. Stowe D. F. (1998). Prior preconditioning by ischemia or sevoflurane improves cardiac work per oxygen use in isolated guinea pig hearts after global ischemia. Adv. Exp. Med. Biol. 454, 533–542 10.1007/978-1-4615-4863-8_64 9889933

[B114] Novalija E. Varadarajan S. G. Camara A. K. An J. Chen Q. Riess M. L. (2002). Anesthetic preconditioning: triggering role of reactive oxygen and nitrogen species in isolated hearts. Am. J. Physiol. Heart Circ. Physiol. 283, H44–H52 10.1152/ajpheart.01056.2001 12063273

[B115] Olson J. Yan Y. Kriegel A. Bai X. Liang M. Bosnjak Z. (2013). miR-21 knockdown attenuates the cardioprotective effects of isoflurane. FASEB J. 27:lb679

[B116] Onishi A. Miyamae M. Kaneda K. Kotani J. Figueredo V. M. (2012). Direct evidence for inhibition of mitochondrial permeability transition pore opening by sevoflurane preconditioning in cardiomyocytes: comparison with cyclosporine A. Eur. J. Pharmacol. 675, 40–46 10.1016/j.ejphar.2011.11.040 22166375

[B117] O'Rourke B. (2004). Evidence for mitochondrial K^+^ channels and their role in cardioprotection. Circ. Res. 94, 420–432 10.1161/01.RES.0000117583.66950.43 15001541 PMC2712129

[B118] Ozcan C. Bienengraeber M. Dzeja P. P. Terzic A. (2002). Potassium channel openers protect cardiac mitochondria by attenuating oxidant stress at reoxygenation. Am. J. Physiol. Heart Circ. Physiol. 282, H531–H539 10.1152/ajpheart.00552.2001 11788400

[B119] Ozcan C. Palmeri M. Horvath T. L. Russell K. S. Russell R. R. 3rd. (2013). Role of uncoupling protein 3 in ischemia-reperfusion injury, arrhythmias, and preconditioning. Am. J. Physiol. Heart Circ. Physiol. 304, H1192–H1200 10.1152/ajpheart.00592.2012 23457013 PMC3652089

[B120] Pagel P. S. (2008). Postconditioning by volatile anesthetics: salvaging ischemic myocardium at reperfusion by activation of prosurvival signaling. J. Cardiothorac. Vasc. Anesth. 22, 753–765 10.1053/j.jvca.2008.03.005 18922439

[B121] Pagel P. S. Krolikowski J. G. Neff D. A. Weihrauch D. Bienengraeber M. Kersten J. R. (2006). Inhibition of glycogen synthase kinase enhances isoflurane-induced protection against myocardial infarction during early reperfusion *in vivo*. Anesth. Analg. 102, 1348–1354 10.1213/01.ane.0000202379.61338.37 16632807

[B122] Pagliaro P. Moro F. Tullio F. Perrelli M. G. Penna C. (2011). Cardioprotective pathways during reperfusion: focus on redox signaling and other modalities of cell signaling. Antioxid. Redox Signal. 14, 833–850 10.1089/ars.2010.3245 20649460

[B123] Pain T. Yang X. M. Critz S. D. Yue Y. Nakano A. Liu G. S. (2000). Opening of mitochondrial K_ATP_ channels triggers the preconditioned state by generating free radicals. Circ. Res. 87, 460–466 10.1161/01.RES.87.6.460 10988237

[B124] Penta de Peppo A. Polisca P. Tomai F. De Paulis R. Turani F. Zupancich E. (1999). Recovery of LV contractility in man is enhanced by preischemic administration of enflurane. Ann. Thorac. Surg. 68, 112–118 10.1016/S0003-4975(99)00466-X 10421125

[B125] Piriou V. Chiari P. Gateau-Roesch O. Argaud L. Muntean D. Salles D. (2004). Desflurane-induced preconditioning alters calcium-induced mitochondrial permeability transition. Anesthesiology 100, 581–588 10.1097/00000542-200403000-00018 15108972

[B126] Porter K. Medford H. M. McIntosh C. M. Marsh S. A. (2012). Cardioprotection requires flipping the ‘posttranslational modification’ switch. Life Sci. 90, 89–98 10.1016/j.lfs.2011.10.026 22154907

[B127] Pravdic D. Hirata N. Barber L. Sedlic F. Bosnjak Z. J. Bienengraeber M. (2012). Complex I and ATP synthase mediate membrane depolarization and matrix acidification by isoflurane in mitochondria. Eur. J. Pharmacol. 690, 149–157 10.1016/j.ejphar.2012.07.003 22796646 PMC3653412

[B128] Pravdic D. Mio Y. Sedlic F. Pratt P. F. Warltier D. C. Bosnjak Z. J. (2010). Isoflurane protects cardiomyocytes and mitochondria by immediate and cytosol-independent action at reperfusion. Br. J. Pharmacol. 160, 220–232 10.1111/j.1476-5381.2010.00698.x 20423337 PMC2874845

[B129] Pravdic D. Sedlic F. Mio Y. Vladic N. Bienengraeber M. Bosnjak Z. J. (2009). Anesthetic-induced preconditioning delays opening of mitochondrial permeability transition pore via protein Kinase C-epsilon-mediated pathway. Anesthesiology 111, 267–274 10.1097/ALN.0b013e3181a91957 19568162 PMC2744603

[B130] Qian L. P. Zhu S. S. Cao J. L. Zeng Y. M. (2005). Isoflurane preconditioning protects against ischemia-reperfusion injury partly by attenuating cytochrome c release from subsarcolemmal mitochondria in isolated rat hearts. Acta Pharmacol. Sin. 26, 813–820 10.1111/j.1745-7254.2005.00117.x 15960887

[B131] Qiao S. Xie H. Wang C. Wu X. Liu H. Liu C. (2013). Delayed anesthetic preconditioning protects against myocardial infarction via activation of nuclear factor-kappaB and upregulation of autophagy. J. Anesth. 27, 251–260 10.1007/s00540-012-1494-3 23143013

[B132] Queliconi B. B. Wojtovich A. P. Nadtochiy S. M. Kowaltowski A. J. Brookes P. S. (2011). Redox regulation of the mitochondrial K_ATP_ channel in cardioprotection. Biochim. Biophys. Acta 1813, 1309–1315 10.1016/j.bbamcr.2010.11.005 21094666 PMC3109179

[B133] Ramakrishna H. Rehfeldt K. H. Pajaro O. E. (2014). Heart transplantation- anesthetic pharmacology and perioperative considerations. Curr. Clin. Pharmacol. [Epub ahead of print]. 10.2174/1574884709666140212104958 25985820

[B134] Raphael J. Abedat S. Rivo J. Meir K. Beeri R. Pugatsch T. (2006). Volatile anesthetic preconditioning attenuates myocardial apoptosis in rabbits after regional ischemia and reperfusion via Akt signaling and modulation of Bcl-2 family proteins. J. Pharmacol. Exp. Ther. 318, 186–194 10.1124/jpet.105.100537 16551837

[B135] Raphael J. Rivo J. Gozal Y. (2005). Isoflurane-induced myocardial preconditioning is dependent on phosphatidylinositol-3-kinase/Akt signalling. Br. J. Anaesth. 95, 756–763 10.1093/bja/aei264 16286350

[B136] Redel A. Lange M. Jazbutyte V. Lotz C. Smul T. M. Roewer N. (2008). Activation of mitochondrial large-conductance calcium-activated K^+^ channels via protein kinase A mediates desflurane-induced preconditioning. Anesth. Analg. 106, 384–391 10.1213/ane.0b013e318160650f 18227289

[B137] Riess M. L. Camara A. K. Chen Q. Novalija E. Rhodes S. S. Stowe D. F. (2002a). Altered NADH and improved function by anesthetic and ischemic preconditioning in guinea pig intact hearts. Am. J. Physiol. Heart Circ. Physiol. 283, H53–H60 10.1152/ajpheart.01057.2001 12063274

[B138] Riess M. L. Camara A. K. Heinen A. Eells J. T. Henry M. M. Stowe D. F. (2008a). KATP channel openers have opposite effects on mitochondrial respiration under different energetic conditions. J. Cardiovasc. Pharmacol. 51, 483–491 10.1097/FJC.0b013e31816bf4a4 18437094 PMC3010203

[B139] Riess M. L. Camara A. K. Novalija E. Chen Q. Rhodes S. S. Stowe D. F. (2002b). Anesthetic preconditioning attenuates mitochondrial Ca^2+^ overload during ischemia in Guinea pig intact hearts: reversal by 5-hydroxydecanoic acid. Anesth. Analg. 95, 1540–1546 10.1097/00000539-200212000-00013 12456413

[B140] Riess M. L. Costa A. D. Carlson R. Jr. Garlid K. D. Heinen A. Stowe D. F. (2008b). Differential increase of mitochondrial matrix volume by sevoflurane in isolated cardiac mitochondria. Anesth. Analg. 106, 1049–1055 10.1213/ane.0b013e318167875e 18349172

[B141] Riess M. L. Eells J. T. Kevin L. G. Camara A. K. Henry M. M. Stowe D. F. (2004a). Attenuation of mitochondrial respiration by sevoflurane in isolated cardiac mitochondria is mediated in part by reactive oxygen species. Anesthesiology 100, 498–505 10.1097/00000542-200403000-00007 15108961

[B142] Riess M. L. Kevin L. G. McCormick J. Jiang M. T. Rhodes S. S. Stowe D. F. (2005). Anesthetic preconditioning: the role of free radicals in sevoflurane-induced attenuation of mitochondrial electron transport in Guinea pig isolated hearts. Anesth. Analg. 100, 46–53 10.1213/01.ANE.0000139346.76784.72 15616050

[B143] Riess M. L. Novalija E. Camara A. K. Eells J. T. Chen Q. Stowe D. F. (2003). Preconditioning with sevoflurane reduces changes in nicotinamide adenine dinucleotide during ischemia-reperfusion in isolated hearts: reversal by 5-hydroxydecanoic acid. Anesthesiology 98, 387–395 10.1097/00000542-200302000-00019 12552198

[B144] Riess M. L. Stowe D. F. Warltier D. C. (2004b). Cardiac pharmacological preconditioning with volatile anesthetics: from bench to bedside? Am. J. Physiol. Heart Circ. Physiol. 286, H1603–H1607 10.1152/ajpheart.00963.2003 15072968

[B145] Rostovtseva T. K. Tan W. Colombini M. (2005). On the role of VDAC in apoptosis: fact and fiction. J. Bioenerg. Biomembr. 37, 129–142 10.1007/s10863-005-6566-8 16167170

[B146] Saotome M. Katoh H. Satoh H. Nagasaka S. Yoshihara S. Terada H. (2005). Mitochondrial membrane potential modulates regulation of mitochondrial Ca2+ in rat ventricular myocytes. Am. J. Physiol. Heart Circ. Physiol. 288, H1820–H1828 10.1152/ajpheart.00589.2004 15563537

[B147] Sedlic F. Pravdic D. Hirata N. Mio Y. Sepac A. Camara A. K. (2010a). Monitoring mitochondrial electron fluxes using NAD(P)H-flavoprotein fluorometry reveals complex action of isoflurane on cardiomyocytes. Biochim. Biophys. Acta 1797, 1749–1758 10.1016/j.bbabio.2010.07.009 20646994 PMC2926458

[B148] Sedlic F. Pravdic D. Ljubkovic M. Marinovic J. Stadnicka A. Bosnjak Z. J. (2009). Differences in production of reactive oxygen species and mitochondrial uncoupling as events in the preconditioning signaling cascade between desflurane and sevoflurane. Anesth. Analg. 109, 405–411 10.1213/ane.0b013e3181a93ad9 19608810 PMC2742556

[B149] Sedlic F. Sepac A. Pravdic D. Camara A. K. Bienengraeber M. Brzezinska A. K. (2010b). Mitochondrial depolarization underlies delay in permeability transition by preconditioning with isoflurane: roles of ROS and Ca2+. Am. J. Physiol. Cell Physiol. 299, C506–C515 10.1152/ajpcell.00006.2010 20519447 PMC2928640

[B150] Shabalina I. G. Nedergaard J. (2011). Mitochondrial (‘mild’) uncoupling and ROS production: physiologically relevant or not? Biochem. Soc. Trans. 39, 1305–1309 10.1042/BST0391305 21936806

[B151] Shimizu S. Matsuoka Y. Shinohara Y. Yoneda Y. Tsujimoto Y. (2001). Essential role of voltage-dependent anion channel in various forms of apoptosis in mammalian cells. J. Cell Biol. 152, 237–250 10.1083/jcb.152.2.237 11266442 PMC2199613

[B152] Shimizu S. Narita M. Tsujimoto Y. (1999). Bcl-2 family proteins regulate the release of apoptogenic cytochrome c by the mitochondrial channel VDAC. Nature 399, 483–487 10.1038/20959 10365962

[B153] Shoshan-Barmatz V. Ben-Hail D. (2012). VDAC, a multi-functional mitochondrial protein as a pharmacological target. Mitochondrion 12, 24–34 10.1016/j.mito.2011.04.001 21530686

[B154] Stadnicka A. Marinovic J. Bienengraeber M. Bosnjak Z. J. (2006). Impact of *in vivo* preconditioning by isoflurane on adenosine triphosphate-sensitive potassium channels in the rat heart: lasting modulation of nucleotide sensitivity during early memory period. Anesthesiology 104, 503–510 10.1097/00000542-200603000-00018 16508398

[B155] Steenbergen C. Murphy E. Levy L. London R. E. (1987). Elevation in cytosolic free calcium concentration early in myocardial ischemia in perfused rat heart. Circ. Res. 60, 700–707 10.1161/01.RES.60.5.700 3109761

[B156] Steenbergen C. Murphy E. Watts J. A. London R. E. (1990). Correlation between cytosolic free calcium, contracture, ATP, and irreversible ischemic injury in perfused rat heart. Circ. Res. 66, 135–146 10.1161/01.RES.66.1.135 2295135

[B157] Stowe D. F. Camara A. K. (2009). Mitochondrial reactive oxygen species production in excitable cells: modulators of mitochondrial and cell function. Antioxid. Redox Signal. 11, 1373–1414 10.1089/ars.2008.2331 19187004 PMC2842133

[B158] Stowe D. F. Kevin L. G. (2004). Cardiac preconditioning by volatile anesthetic agents: a defining role for altered mitochondrial bioenergetics. Antioxid. Redox Signal. 6, 439–448 10.1089/152308604322899512 15025946

[B159] Stumpner J. Lange M. Beck A. Smul T. M. Lotz C. A. Kehl F. (2012a). Desflurane-induced post-conditioning against myocardial infarction is mediated by calcium-activated potassium channels: role of the mitochondrial permeability transition pore. Br. J. Anaesth. 108, 594–601 10.1093/bja/aer496 22315330

[B160] Stumpner J. Smul T. M. Redel A. Hilz T. Tischer-Zeitz T. Eisenbarth H. (2012b). Desflurane-induced and ischaemic postconditioning against myocardial infarction are mediated by Pim-1 kinase. Acta Anaesthesiol. Scand. 56, 904–913 10.1111/j.1399-6576.2012.02657.x 22385356

[B161] Szabo I. Zoratti M. (1993). The mitochondrial permeability transition pore may comprise VDAC molecules. I. Binary structure and voltage dependence of the pore. FEBS Lett. 330, 201–205 10.1016/0014-5793(93)80273-W 7689983

[B162] Tanaka K. Ludwig L. M. Kersten J. R. Pagel P. S. Warltier D. C. (2004a). Mechanisms of cardioprotection by volatile anesthetics. Anesthesiology 100, 707–721 10.1097/00000542-200403000-00035 15108989

[B163] Tanaka K. Ludwig L. M. Krolikowski J. G. Alcindor D. Pratt P. F. Kersten J. R. (2004b). Isoflurane produces delayed preconditioning against myocardial ischemia and reperfusion injury: role of cyclooxygenase-2. Anesthesiology 100, 525–531 10.1097/00000542-200403000-00010 15108964

[B164] Tanaka K. Weihrauch D. Kehl F. Ludwig L. M. Ladisa J. F. Jr. Kersten J. R. (2002). Mechanism of preconditioning by isoflurane in rabbits: a direct role for reactive oxygen species. Anesthesiology 97, 1485–1490 10.1097/00000542-200212000-00021 12459675

[B165] Tanguay M. Blaise G. Dumont L. Beique G. Hollmann C. (1991). Beneficial effects of volatile anesthetics on decrease in coronary flow and myocardial contractility induced by oxygen-derived free radicals in isolated rabbit hearts. J. Cardiovasc. Pharmacol. 18, 863–870 10.1097/00005344-199112000-00012 1725899

[B166] Tikunov A. Johnson C. B. Pediaditakis P. Markevich N. Macdonald J. M. Lemasters J. J. (2010). Closure of VDAC causes oxidative stress and accelerates the Ca^2+^-induced mitochondrial permeability transition in rat liver mitochondria. Arch. Biochem. Biophys. 495, 174–181 10.1016/j.abb.2010.01.008 20097153 PMC2855314

[B167] Tonkovic-Capin M. Gross G. J. Bosnjak Z. J. Tweddell J. S. Fitzpatrick C. M. Baker J. E. (2002). Delayed cardioprotection by isoflurane: role of K_ATP_ channels. Am. J. Physiol. Heart Circ. Physiol. 283, H61–H68 10.1152/ajpheart.01040.2001 12063275

[B168] Tullio F. Angotti C. Perrelli M. G. Penna C. Pagliaro P. (2013). Redox balance and cardioprotection. Basic Res. Cardiol. 108:392 10.1007/s00395-013-0392-7 24158692

[B169] Vander Heiden M. G. Chandel N. S. Li X. X. Schumacker P. T. Colombini M. Thompson C. B. (2000). Outer mitochondrial membrane permeability can regulate coupled respiration and cell survival. Proc. Natl. Acad. Sci. U.S.A. 97, 4666–4671 10.1073/pnas.090082297 10781072 PMC18290

[B170] Van der Linden P. J. Daper A. Trenchant A. De Hert S. G. (2003). Cardioprotective effects of volatile anesthetics in cardiac surgery. Anesthesiology 99, 516–517 10.1097/00000542-200308000-00048 12883436

[B171] Vinnakota K. C. Dash R. K. Beard D. A. (2011). Stimulatory effects of calcium on respiration and NAD(P)H synthesis in intact rat heart mitochondria utilizing physiological substrates cannot explain respiratory control *in vivo*. J. Biol. Chem. 286, 30816–30822 10.1074/jbc.M111.242529 21757763 PMC3162442

[B172] Walters A. M. Porter G. A. Jr. Brookes P. S. (2012). Mitochondria as a drug target in ischemic heart disease and cardiomyopathy. Circ. Res. 111, 1222–1236 10.1161/CIRCRESAHA.112.265660 23065345 PMC3507431

[B173] Wang C. Neff D. A. Krolikowski J. G. Weihrauch D. Bienengraeber M. Warltier D. C. (2006a). The influence of B-cell lymphoma 2 protein, an antiapoptotic regulator of mitochondrial permeability transition, on isoflurane-induced and ischemic postconditioning in rabbits. Anesth. Analg. 102, 1355–1360 10.1213/01.ane.0000202463.28618.64 16632808

[B174] Wang C. Weihrauch D. Schwabe D. A. Bienengraeber M. Warltier D. C. Kersten J. R. (2006b). Extracellular signal-regulated kinases trigger isoflurane preconditioning concomitant with upregulation of hypoxia-inducible factor-1alpha and vascular endothelial growth factor expression in rats. Anesth. Analg. 103, 281–288 10.1213/01.ane.0000226094.94877.98 16861403

[B175] Wang L. Cherednichenko G. Hernandez L. Halow J. Camacho S. A. Figueredo V. (2001). Preconditioning limits mitochondrial Ca^2+^ during ischemia in rat hearts: role of K_ATP_ channels. Am. J. Physiol. Heart Circ. Physiol. 280, H2321–H2328 11299237 10.1152/ajpheart.2001.280.5.H2321

[B176] Warltier D. C. Al-Wathiqui M. H. Kampine J. P. Schmeling W. T. (1988). Recovery of contractile function of stunned myocardium in chronically instrumented dogs is enhanced by halothane or isoflurane. Anesthesiology 69, 552–565 10.1097/00000542-198810000-00016 3177915

[B177] Weber N. C. Schlack W. (2008). Inhalational anaesthetics and cardioprotection. Handb. Exp. Pharmacol. 182, 187–207 10.1007/978-3-540-74806-9_9 18175092

[B178] Weihrauch D. Krolikowski J. G. Bienengraeber M. Kersten J. R. Warltier D. C. Pagel P. S. (2005). Morphine enhances isoflurane-induced postconditioning against myocardial infarction: the role of phosphatidylinositol-3-kinase and opioid receptors in rabbits. Anesth. Analg. 101, 942–949 10.1213/01.ane.0000171931.08371.a2 16192500

[B179] Wick M. J. Mihic S. J. Ueno S. Mascia M. P. Trudell J. R. Brozowski S. J. (1998). Mutations of gamma-aminobutyric acid and glycine receptors change alcohol cutoff: evidence for an alcohol receptor? Proc. Natl. Acad. Sci. U.S.A. 95, 6504–6509 10.1073/pnas.95.11.6504 9600996 PMC27833

[B180] Wojtovich A. P. Williams D. M. Karcz M. K. Lopes C. M. Gray D. A. Nehrke K. W. (2010). A novel mitochondrial K_ATP_ channel assay. Circ. Res. 106, 1190–1196 10.1161/CIRCRESAHA.109.215400 20185796 PMC2857559

[B181] Wong R. Aponte A. M. Steenbergen C. Murphy E. (2010). Cardioprotection leads to novel changes in the mitochondrial proteome. Am. J. Physiol. Heart Circ. Physiol. 298, H75–H91 10.1152/ajpheart.00515.2009 19855063 PMC2806145

[B182] Wu W. Zhou X. Liu P. Fei W. Li L. Yun H. (2014). Isoflurane reduces hypoxia/reoxygenation-induced apoptosis and mitochondrial permeability transition in rat primary cultured cardiocytes. BMC Anesthesiol. 14:17 10.1186/1471-2253-14-17 24612850 PMC3975578

[B183] Yang M. Camara A. K. Wakim B. T. Zhou Y. Gadicherla A. K. Kwok W. M. (2012). Tyrosine nitration of voltage-dependent anion channels in cardiac ischemia-reperfusion: reduction by peroxynitrite scavenging. Biochim. Biophys. Acta 1817, 2049–2059 10.1016/j.bbabio.2012.06.004 22709907 PMC3985433

[B184] Yin C. Salloum F. N. Kukreja R. C. (2009). A novel role of microRNA in late preconditioning: upregulation of endothelial nitric oxide synthase and heat shock protein 70. Circ. Res. 104, 572–575 10.1161/CIRCRESAHA.108.193250 19213952 PMC3359791

[B185] Zalk R. Israelson A. Garty E. S. Azoulay-Zohar H. Shoshan-Barmatz V. (2005). Oligomeric states of the voltage-dependent anion channel and cytochrome c release from mitochondria. Biochem. J. 386, 73–83 10.1042/BJ20041356 15456403 PMC1134768

[B186] Zaugg M. Lucchinetti E. Garcia C. Pasch T. Spahn D. R. Schaub M. C. (2003a). Anaesthetics and cardiac preconditioning. Part II. Clinical implications. Br. J. Anaesth. 91, 566–576 10.1093/bja/aeg206 14504160

[B187] Zaugg M. Lucchinetti E. Uecker M. Pasch T. Schaub M. C. (2003b). Anaesthetics and cardiac preconditioning. Part I. Signalling and cytoprotective mechanisms. Br. J. Anaesth. 91, 551–565 10.1093/bja/aeg205 14504159

[B188] Zaugg M. Schaub M. C. (2003). Signaling and cellular mechanisms in cardiac protection by ischemic and pharmacological preconditioning. J. Muscle Res. Cell Motil. 24, 219–249 10.1023/A:1026021430091 14609033

[B189] Zhao Z. Q. Nakamura M. Wang N. P. Wilcox J. N. Shearer S. Ronson R. S. (2000). Reperfusion induces myocardial apoptotic cell death. Cardiovasc. Res. 45, 651–660 10.1016/S0008-6363(99)00354-5 10728386

[B190] Zhu J. Rebecchi M. J. Tan M. Glass P. S. Brink P. R. Liu L. (2010). Age-associated differences in activation of Akt/GSK-3beta signaling pathways and inhibition of mitochondrial permeability transition pore opening in the rat heart. J. Gerontol. A Biol. Sci. Med. Sci. 65, 611–619 10.1093/gerona/glq035 20427381

[B191] Zima A. V. Blatter L. A. (2006). Redox regulation of cardiac calcium channels and transporters. Cardiovasc. Res. 71, 310–321 10.1016/j.cardiores.2006.02.019 16581043

